# Fatigue damage assessment of complex railway turnout crossings via Peridynamics-based digital twin

**DOI:** 10.1038/s41598-022-18452-w

**Published:** 2022-08-23

**Authors:** Mehmet Hamarat, Mayorkinos Papaelias, Sakdirat Kaewunruen

**Affiliations:** 1 Birmingham Centre for Railway Research and Education, School of Engineering, The University of Birmingham, Birmingham, B15 2TT UK; 2 School of Metallurgy and Materials, The University of Birmingham, Birmingham, B15 2TT UK

**Keywords:** Civil engineering, Mechanical engineering, Engineering

## Abstract

Railway turnouts are essential in the train traffic route management for modern railways. Despite significant devotion to railway turnout research, one of their most common failures has not been thoroughly investigated, which is a fatigue over the turnout crossing nose. At the crossings, wheel-rail discontinuity imparts high-frequency high-magnitude forces, which are the source of fatigue failure over the crossing nose. In this study, a novel approach built on “Peridynamics” (PD) has been developed to obtain new insights into the fatigue cracks. A recent approach using “crack on mid-plane” has also been employed in this study to enhance the limited capability of Peridynamics. This paper is the world’s first to investigate fatigue failures over a crossing nose from fracture mechanics perspective. This paper also introduces a novel adaptive time-mapping method as an alternative to earlier time-mapping methods for fatigue models proposed in the open literature. The new model has been verified against both Finite Element Method and experimental data. It reveals that our new approach can simulate fatigue damage, particularly in mode I crack propagation. The study has provided important insights on the fatigue crack development, which is not possible before by existing Peridynamics fatigue model. The new approach on the basis of “adaptive time-mapping” and “crack on mid-plane” is demonstrated to be effective and efficient in PD simulations.

## Introduction

Fatigue damage over a crossing nose of a turnout is a well-known problem among railway infrastructure managers. It emerges from high-frequency high-magnitude wheel forces, namely impact forces^1–4^. A crossing nose is located at the intersection point of two rails of a turnout and ensures a clear passage for a rolling stock that is diverted from one route to another route via turnout switches. A large number of turnout research have increased in last few decades^5–13^ and many of them have investigated the causes or effects of the damages associated with fatigue over a crossing nose^2,3,14–21^. The main motivation behind those research was to mitigate considerably high maintenance and procurement costs of turnout systems^22,23^. In addition, those research have paved the way for the digital twin concept. Digital twin is basically the digital representation of the physical objects or systems by considering their pyhsical and functional characteristics in order to estimate their behaviour at present or in future^24^. In recent years, the concept of digital twin has also attracted many researchers in railway systems, including the ones who are interested in railway turnouts^5,25,26^.

Over the last two decades, many researchers have been trying to numerically simulate cracks as well as fatigue cracks^27–32^. A large portion of those studies focused on establishing governing equations via small-scale geometries. Complex crack behaviour and computational cost are two main challenges in crack simulations. Hence, simulations of large-scale models seem to be rarely demonstrated. In addition to those, fatigue is long-term damage and investigations of long-term damages via numerical tools are also computationally expensive tasks. On railways studies, few attempts of simulating the long-term damages, particularly on crossing noses could be found in the literature^19,20^. In those studies, plastic deformations in 2D were of interest.

The first numerical method that comes into mind is the well-known Finite Element Method (FEM). In the theory of FEM, displacements of nodes of a discretized body are evaluated and interpreted within the rules of classical continuum theory (CCT). Even though FEM is a promising tool to solve many multi-disciplinary challenges, it presents flaws while solving crack-oriented problems^33^. The fundamental differential equations of CCT lead to inconsistencies when the deformations are large or produce discontinuities in the body. To get around those problems associated with differential equations, several sophisticated remedies could be found within the open literature^27,28^. Nonetheless, in general, expert researchers can utilise those solutions. Here, it is noteworthy that the other methods involving partial derivatives, most of which are based on FEM, will not be discussed here since they also seek sophisticated solutions to overcome the drawbacks associated with the partial derivatives. In other words, any methods using external criteria are perceived as forced solutions and has been treated similar to FEM here.

An effective, inherent solution to crack-oriented problems is Silling’s theory of Peridynamics (PD)^34^. In PD, crack initiation and propagation occur naturally owing to the volume integrals in the definition of the problem and there is no need to formulate crack behaviour to avoid common problems in CCT^33^. Consequently, various crack problems from stationary single crack to dynamic multiple cracks as well as fatigue cracks were examined in numerous studies earlier^35–40^. Furthermore, PD has also been found to be suitable to utilize many material models such as elastic, plastic, composites, polycrystalline, etc^41–45^.

Interestingly, the application of PD theory in railways is still relatively rare. The studies in^46^ and^47^ could be accepted as pioneering works. In both studies they have applied the fatigue model as proposed in^48^. Meanwhile in^46^, the rail squats were examined in terms of crack propagation whereas crack initiation on a rail surface were assessed in^47^. Both studies assumed that quasi-static non-cyclic loading would be sufficient to validate the fatigue model that was subjected to cyclic loadings. Furthermore, those studies ignored the question about how to associate the damage value with the crack formation in Peridynamics. They preferred a common method of “nodal damage value” that gives the ratio of broken bonds over total bonds of a point. However, “nodal damage value” is normally insufficient to show whether there is a crack or not. That is because there is no consensus on the decision of nodal damage values. Even small differences influence the crack behaviour and result in dissimilar outcomes. Similarly, in^48^, it was pointed out that it was a challenge to determine a correct nodal damage value enabling the phase transformation from crack initiation to crack propagation. Therefore, available past studies did not consider both phases, together. To solve those problems originating from nodal damage value, authors previously developed a novel method, namely “crack on mid-plane”^49^. The method enables tracking crack formations and more importantly, produces a single outcome for PD simulations. Furthermore, it inherently allows phase transformation from crack initiation to propagation. Here, it is noteworthy that the method of “crack on mid-plane” is different in comparison to critical plane approaches in continuum theories. Critical plane approaches try to define the crack orientation and formation^50^ on the contrary to Peridynamics where a crack occurs inherently. The method of “crack on mid-plane” is a way of assesing the damage of the non-local structure of PD nodes.

The computational cost of fatigue simulations in PD can be extremely high. Hence, it is imperative to use time-mapping methods^48^. Nevertheless, time mapping may result in numerical instabilities such as breaking more bonds in comparison to a solution without time-mapping. In^51^, it was proposed, as a remedy for the numerical instability, to use two constants (minimum and maximum damage values) in order to regulate outcomes of simulation with a linear time-mapping. In^51^, if the time-mapping constant causes more damage than the maximum damage level, then they reduce the number of cycles by 0.5 and repeat the simulation. Conversely, they skipped the solution process and expected the same outcome if the damage is lower than the minimum damage. Even though the method in^51^ ensured relative stability, it introduced new challenges such as determination of $D_{\min }$ and $D_{\max }$. Furthermore, it might cause longer simulations when crack speeds escalate. Another solution was presented in^52^, which proposed to solve bond life issue analytically. Nevertheless, its analytical solution was not validated for the crack initiation part. Furthermore, no ground was mathematically provided for the assumption of constant cyclic strain in the crack propagation part. It is essentially crucial because the analytical method seems to be against the characteristics of the crack propagation part of the fatigue model in^48^ which acknowledges the strain variations under cycling loadings at the core.

The aim of this study is to investigate the dynamic fragility of a crossing nose of railway turnouts in terms of fatigue crack by utilizing the “crack on mid-plane” method and PD theory. The integrated approach is highly novel and innovative since it is the first time that the fatigue damage over a crossing nose could be thoroughly investigated from fracture mechanics perspective. Furthermore, the improvement of the conventional fatigue model^48^ with “crack on mid-plane” allows to track fatigue crack development and for the first time, consider phase transformation from initiation to propagation beyond the state of the art methods in Peridynamics. In addition, a novel time-mapping method for fatigue calculations is proposed in this study. Time-mappings are utilized to speed up the calculations. As contrary to^46,47^, the validation process in this study also involves the cyclic loadings. The paper begins by introducing PD theory, state-based PD, the fatigue model, “crack on mid-plane” and adaptive time-mappings methods; carrying on demonstrating the PD models in details, the validation of the models, and ends up with the results, discussions and conclusions.

## Methodology

### Background for a state-based Peridynamics theory

**Figure 1:**
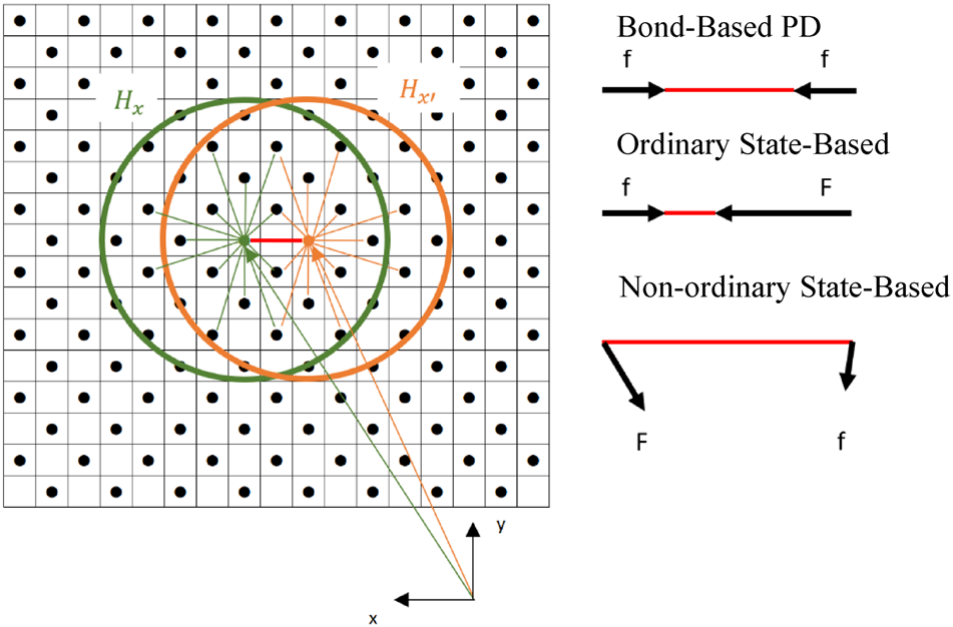
An illustration of PD theory (left). The illustrations of bond interactions in accordance to different Peridynamic theories (right).

PD discretizes a body into the points occupying volume in space and governs the relations between each point and its neighbours within a certain distance^33^. In other words, each point in the body is linked to its neighbours, by so-called bonds and the behaviour of each point is defined by the resultant force of those bonds. To calculate general behaviour, a function that maps every point in the body from undeformed configuration to deformed configuration is integrated over the volume of the body. Due to integration operation, PD is able to deal with discontinuities inherently^34^. Nevertheless, it is computationally expensive due to its non-local structure. It is noteworthy that each point or node in PD considers not only adjacent points but also any points in a certain distance (so-called horizon) as neighbours. Therefore, a trade-off must be considered for any application of PD. The original version of PD is called ‘Bond-based Peridynamics’ theory and assumes that any two points apply the same magnitude forces in opposite directions on the same axis. Thus, it is found to be valid for certain Poisson’s ratios. Further information could be found in^34,53^. To overcome the bespoken limitations in terms of Poisson’s ratio, a more generalized version of PD theory, a state-based PD theory, has been offered^53^. The ‘state’ is a specifically defined mathematical tensor that specifies the relation between undeformed and deformed configurations. The state-based theory can be further categorized into two subsections as ordinary and non-ordinary state-based theories. In ordinary state-based PD, the forces are collinear in opposite directions with different magnitudes. On the contrary, non-ordinary state-based PD involves arbitrary forces with various magnitudes. It should be reminded that non-ordinary state-based models are also called “correspondence models” which yield the same results with the classical material models^53,54^. In other words, non-ordinary state-based PD produces outcomes that can be expressed in terms of stresses. Even though non-ordinary models sound very effective, they suffer from instability^55^. An illustration of PD is depicted in Fig. 1.

### Ordinary state-based Peridynamics theory

Ordinary state-based Peridynamics (OSBP) is a generalized version of the original PD formula. OSBP maintains its validity for any given Poisson’s ratio and discriminates the forces acting on two neighbouring points. In PD theory, the displacements of neighbour pairs (x, x′) can be written as are shown in Eq. (1).

1 $${\mathbf{u}} = {\mathbf{y}} - {\mathbf{x}} , {\mathbf{u}}{^{\prime}} = {\mathbf{y}}{^{\prime}} - {\mathbf{x}}{^{\prime}}$$

Length and relative displacement of a bond is expressed in Eq. (2) as

2 $$\begin{gathered} {\varvec{\xi}} = {\mathbf{x}}{^{\prime}} - {\mathbf{x}} \hfill \\ {\varvec{\eta}} = {\mathbf{u^{\prime}}} - {\mathbf{u}} \hfill \\ \end{gathered}$$

Then, bond stretch can be written as is in Eq. (3).

3 $$s = \frac{{\left| {\varvec{\eta}} \right|}}{{\left| {\varvec{\xi}} \right|}}$$

In most PD studies, damage of a bond is defined by a condition stating “a bond will break if it stretches further than the critical stretch value” (Eq. (4), where $i,j$ are bond identification indexes). It is worth mentioning that there is also an energy-based condition which is expressed as: “A bond will break if the energy stored in the relevant bond exceeds a certain threshold”. Energy-based failure criterion can be found in^56^. Here, it is neglected as it reflects a similar concept with the critical stretch criterion. In other words, energy and critical stretch can be used interchangeably in most cases having link to a correlation between energy and deformation^57^.

4 $$w\left( {\varvec{\xi}} \right) = \left\{ {\begin{array}{*{20}c} 1 \\ 0 \\ \end{array} { }\begin{array}{*{20}c} {if{ }s_{ij} < s_{c} } \\ {if{ }s_{ij} > s_{c} } \\ \end{array} } \right.$$

In the state-based PD, a state is basically a matrix that stores the information of all points in the body for a given parameter. It is a mathematically defined specific tensor that can store different types of information^53^. The concept of the state is a necessity to introduce discontinuities and large deformations. For instance, a vector state is similar to a second-order tensor as they both map vectors into vectors^47^. Differently, a vector state is capable of considering discontinuities and expressing non-linear relations in contrast to a second-order tensor. Note that, a vector state has infinite dimensions whereas a tensor has only 9.

In state-based PD, a deformation state is defined by relative distance between the points. Equation (5) denotes the deformation state of point x.

5 $${\mathbf{\underline {Y} }}_{{{ }x_{i} }} = \left\{ {\begin{array}{*{20}c} {y_{1} - y_{i} } \\ \ldots \\ {y_{n} - y_{i} } \\ \end{array} } \right\}$$

Likewise, a vector state, ${\mathbf{\underline {X} }}$, of the reference position and a vector state of deformation can also be expressed by the notations as below in Eqs. (6) and (7).

6 $${\mathbf{\underline {X} }}\left\langle {{\mathbf{x^{\prime}}} - {\mathbf{x}}} \right\rangle_{ } = {\mathbf{x^{\prime}}} - {\mathbf{x}}$$

7 $${\mathbf{\underline {Y} }}\left\langle {{\mathbf{x^{\prime}}} - {\mathbf{x}}} \right\rangle_{ } = {\mathbf{y^{\prime}}} - {\mathbf{y}}$$

PD defines the motion of a body by interpreting deformations those result in forces. Hence, the force state is dependent on deformations as shown in Eq. (8).

8 $${\mathbf{\underline {T} }}\left( {{\mathbf{x}},t} \right) = {\mathbf{\underline {T} }}\left( {{\mathbf{\underline {Y} }}\left( {{\mathbf{x}},t} \right)} \right)$$

In PD, a force state vector consists of elements called “force density vectors”(Eq. 9). That is because a point is subjected to collective interactions between the point and its neighbours.

9 $${\mathbf{\underline {T} }}_{{{ }x_{i} }} = \left\{ {\begin{array}{*{20}c} {t_{1} - t_{i} } \\ \ldots \\ {t_{n} - y_{i} } \\ \end{array} } \right\}$$

In ordinary state-based PD, force density vectors are not equal to each other (as shown in Eqs. (10) and (11)).

10 $${\mathbf{t}}\left( {{\mathbf{u^{\prime}}} - {\mathbf{u}},{\mathbf{x^{\prime}}} - {\mathbf{x}},t} \right) = {\mathbf{\underline {T} }}\left( {{\mathbf{x}},t} \right) < {\mathbf{x^{\prime}}} - {\mathbf{x}} \ge = \frac{1}{2}A.\frac{{{\mathbf{y^{\prime}}} - {\mathbf{y}}}}{{\left| {{\mathbf{y^{\prime}}} - {\mathbf{y}}} \right|}}$$

11 $${\mathbf{t}}\left( {{\mathbf{u}} - {\mathbf{u}}{^{\prime}},{\mathbf{x}} - {\mathbf{x}}{^{\prime}},t} \right) = {\mathbf{\underline {T} }}\left( {{\mathbf{x}}{^{\prime}},t} \right) < {\mathbf{x}} - {\mathbf{x^{\prime}}} \ge = - \frac{1}{2}B.\frac{{{\mathbf{y^{\prime}}} - {\mathbf{y}}}}{{\left| {{\mathbf{y^{\prime}}} - {\mathbf{y}}} \right|}}$$

where A, B are parameters dependent on the material properties, deformation field and so on. The equations point out that the forces in OSBPD are collinear with different magnitudes. It is worth mentioning the equality of A to B in BBPD. Finally, the equation of motion in OSBPD is given as

12 $$\rho \left( {\mathbf{x}} \right).{\mathbf{\ddot{u}}}\left( {{\mathbf{x}},t} \right) = \int\limits_{{H_{{\mathbf{x}}} }} {\left( {{\mathbf{\underline {T} }}\left( {{\mathbf{x}},t} \right)\left\langle {{\mathbf{x}}^{\prime} - {\mathbf{x}}} \right\rangle - {\mathbf{\underline {T} }}\left( {{\mathbf{x}}^{\prime},t} \right)\left\langle {{\mathbf{x}} - {\mathbf{x}}^{\prime}} \right\rangle } \right).dV_{{{\mathbf{x}}^{\prime}}} + {\varvec{b}}\left( {{\mathbf{x}},t} \right)}$$

where $\rho$ is density, ${\mathbf{\ddot{u}}}$ is acceleration,$\user2{ b}$ is the external force density, ${\mathbf{\underline {T} }}$ is the force vector state field and $H_{{\mathbf{x}}}$ is horizon, the volume centred at ${\mathbf{x}}$ that contains neighbours of ${\mathbf{x}}$ within a radius of $\delta$**.**

### Fatigue model

In this paper, the fatigue model proposed in^48^ is also preferred. The method assumes that a bond will break after some cyclic strains. The cyclic strain is expressed as shown in Eq. (13),

13 $$\varepsilon_{ij} = \left| {s_{ij}^{ + } - s_{ij}^{ - } } \right|$$

where the positive and negative signs indicate two extreme bond stretches at minimum and maximum loadings. Note that $i,j$ are the indexes to identify the bonds. The fatigue model assumes that each bond has a constant life, $\lambda_{0}$ and $\lambda_{o}$ degrades with a function of strain and cycle ($\lambda_{\varepsilon , N}$). A bond will fail if the condition in Eq. (14) is satisfied.

14 $$\lambda_{0} - \lambda_{{\varepsilon ,{ }N}} = 0$$

For convenience, it is assumed that cyclic strain is independent of N and $\lambda_{0}$ is equal to 1. Then, the above condition can be defined as follows,

15 $$A_{1} .\varepsilon_{1}^{{m_{1} }} .N_{1} = 1{ }$$

where $A_{1} ,m_{1}$ are the material constants extracted from strain-life curves. Equation (15) is proposed for the crack initiation part since it holds no information about crack propagation. For the crack propagation part, it is proposed that there is a bond located at the crack tip and on the verge of breaking as called “the core bond”. When this bond is broken, then crack propagates towards the next core bond. The relation between the core bonds and crack propagation speed is given as

16 $$\frac{{{\text{d}}a}}{{{\text{d}}N}} = A_{2} .\varepsilon_{core}^{{m_{2} }}$$

where $A_{2} ,m_{2}$ are the material constants. Equation (16) is similar to the well-known Paris law for fatigue crack growth.

17 $$\frac{{{\text{d}}a}}{{{\text{d}}N}} = C{\Delta }K^{m}$$

where c, m are the material constants and K is the stress-intensity factor. A comparison between Eqs. (16) and (17) leads to a conclusion so that the exponents must be the same in both equations. Nevertheless, the constants $A_{2} ,C$ are different since the relation between $\varepsilon_{core}$ and stress intensity factor is unknown. To determine the constant $A_{2}$, a simulation must be run with a random $A_{2}^{^{\prime}}$ value. Then, $A_{2}$ value can be calculated via Eq. (18).

18 $$A_{2} = A_{2}^{^{\prime}} .\frac{{\left( {\frac{{{\text{d}}a}}{{{\text{d}}N}}} \right)_{ } }}{{\left( {\frac{{{\text{d}}a}}{{{\text{d}}N}}} \right)^{^{\prime}}_{ } }}$$

Lastly but importantly, as indicated in^48^, the constant $A_{2}$ is influenced by PD parameter of horizon linked to the definition of the core bond. Thus, $A_{2}$ can be scaled with respect to the horizon via Eq. (19).

19 $$A_{2} \left( \delta \right) = \hat{A}_{2} .\delta^{{\frac{{m_{2} - 2}}{2}}}$$

where the constant $\hat{A}_{2}$ is independent of $\delta$.

### The method of crack on mid-plane

The concept of bonds and non-locality in PD provides unique properties that can be considered within the rules of physics rather than mathematics. Hence, this physical meaning of PD could be used to assess the damage of material points in a given body. A general approach to assess damage by PD simulations is “nodal damage value” that gives the number of bonds which must be broken to initiate damage in a given body. Nevertheless, this approach suffers from a lack of methodology to conclude the decision on the magnitude of nodal damage value. In^49^, it was shown that the magnitude of damage value influences crack behaviour considerably. Hence, a methodology independent of nodal damage value was proposed in^49^. The same methodology has been also employed in this study.

According to^49^, a crack would occur when all direct and first-degree indirect bonds between two points are broken. The direct bond is the bond between two points and the first-degree indirect bonds are the bonds that are connected to common neighbours of those two points. Nevertheless, the direct application of the proposed method is assumed to be computationally expensive due to the number of subsets which store information about all bonds. Therefore, the method has been further developed by assuming all direct and indirect bonds pass through a plane between two points. Then, the damage could be found by Eqs. (20) and (21).

20 $${\text{\rm Z}}\left( x \right) = \mathop \smallint \limits_{{\mathcal{P}}}^{ } \left( {1 - w\left( {\left| \xi \right|} \right)} \right){\text{d}}A$$

21 $$\phi \left( x \right),\phi \left( {x{^{\prime}}} \right) = \left\{ {\begin{array}{*{20}l} 1 \hfill & {if\quad {\text{\rm Z}}\left( x \right) = 0{ } \wedge {\text{ {\rm Z}}}\left( {x^{\prime}} \right) = 0} \hfill \\ 0 \hfill & {else} \hfill \\ \end{array} } \right.$$

where ${\text{\rm Z}},P,{\upxi },w,A,\phi ,x,x{^{\prime}}$ are damage function, plane, bond vector, PD influence function, bond area, crack propagation value, point x and its neighbour x’, respectively. The plane is defined at the mid-location between the two-points by Eq. (22), which facilitates the problem significantly and gives a name to the method.

22 $${\varvec{\xi}}{ }.{ }\left( {{ }{\varvec{p}} - {\varvec{p}}_{0} } \right) = 0$$

In Eq. (22), ${\varvec{\xi}},\user2{p }$ and ${\varvec{p}}_{0}$ are the bond vector, the points on the plane and the mid-point between the two points, respectively.

### Adaptive time-mapping

The fatigue model employed in this study is based on two assumptions such that strains on each cycle are the same unless a bond is broken. Likewise, the crack propagation speed on each cycle is also the same during crack propagation from one core bond to another. In^48^, the concept of time mapping was suggested to reduce simulation time with reference to those assumptions. Equations (23) and (24) are the time-mapping equations as proposed in^48^.

23 $$N = t/\tau$$

24 $$N = e^{t/\tau }$$

where $N,t,\tau$ are a number of cycles, time step and mapping constant, respectively. As aforementioned, the life of a bond is a function of cycle. Hence, time-mapping offers that multiple cycles can be used for a given time step instead of a single cycle. For instance, assuming that one cycle takes place in one second and the mapping constant is 0.1, then, the linear time-mapping will produce 10 cycles in one second.

In this study, a new time-mapping approach is proposed. The new method is fully adaptive and changes time-mapping constants throughout the simulation. Hence, it protects the original concept as proposed in^48^. As stated in Eq. (15), the total life is the product of cycle, strain and material constants. When mapping is applicable, Eq. (15) can be written as Eq. (25) as below;

25 $$A_{1} \cdot \varepsilon_{1}^{{m_{1} }} \cdot N_{1} = A_{1} \cdot \varepsilon_{1}^{{m_{1} }} \cdot N \cdot \tau = 1{ }$$

where $N$ is the number of cycles. Here, it should be emphasized that the time-mapping constant,$\tau ,$ is expressed differently but used for the same purpose in Eqs. (23) and (24). If a single bond is to be broken, it is clear that the ideal time-mapping constant will be as follows in Eq. (26).

26 $$\tau_{i} = 1/\left( {A_{1} \cdot \varepsilon_{1}^{{m_{1} }} } \right)$$

Nevertheless, Eq. (26) cannot be applicable for multiple bond-breaking in a given body since the ideal number will be significantly higher and may lead to a failure of all bonds in the following cycle. To consider multiple bond-breaking, the remaining life of bonds must be considered. Hence, Eq. (27) can be written alternatively as

27 $$\tau_{a} = \lambda_{r} \left( t \right)/\left( {A_{1} \cdot \varepsilon_{1}^{{m_{1} }} } \right)$$

where $\lambda_{r} \left( t \right)$ represents the remaining life of a bond. A similar constant can be defined for the crack propagation part as shown in Eq. (28).

28 $$\tau_{a} = \lambda_{r} \left( t \right)/\left( {A_{2} \cdot \varepsilon_{2}^{{m_{2} }} } \right)$$

Then, the adaptive time-mapping constant can be defined as the minimum of adaptive time-mapping constant state (Eq. 29).

29 $$\begin{gathered} \tau_{a} = {\text{floor}}\left( {{\text{min}}\left( { \underline{{{\varvec{\tau}}_{{\varvec{a}}} }} \user2{ }} \right) } \right) if \tau_{a} > 1,\quad \lambda_{r} > 0 \hfill \\ else \ \tau_{a} = 1 \hfill \\ \end{gathered}$$

Following the several test simulations, two more optional conditions are added to reduce simulation time and also to increase the accuracy further. It was found that the code calculates slightly higher cycles due to the “floor” function and the structure of the code, which will be explained in the next section. For instance, there were always some bonds with the remaining lives close to 0. This resulted in $\tau_{a}$ equals 1. In other words, the bonds were broken 1 cycle later. The other factors will be explained in the next section.

The first optional condition is to add a tuning constant, called the “sensitivity” to ignore the remaining life of bonds that are lower than the specified threshold (Eq. 30), which leads to higher accuracy. This will be particularly effective when the life of a bond is close to zero. This constant also eliminates some drawbacks originating from the code structure. The sensitivity constant can be adjusted by running several simulations and comparing results with a reference case that does not utilize time-mapping. The sensitivity constant is a kind of offset value from the reference case. It is worth mentioning that the time-mapping constant goes to zero when strains go to infinity in Eqs. (27) and (28). In other words, the effect of neglecting some bonds that have remaining lives below a threshold will be limited when strains are high.

30 $$\lambda_{r} \left( t \right) > c$$

where c is the threshold for remaining life to include bonds into the calculation. Aside from the sensitivity, linear time-mapping can be combined with adaptive time mapping to obtain the fastest solution in exchange of losing stability at some degree (Eq. 31).

31 $$\tau_{a} = 1/\tau_{linear} if \tau_{a} < \tau_{linear}$$

### PD models and analysis procedure

**Figure 2:**
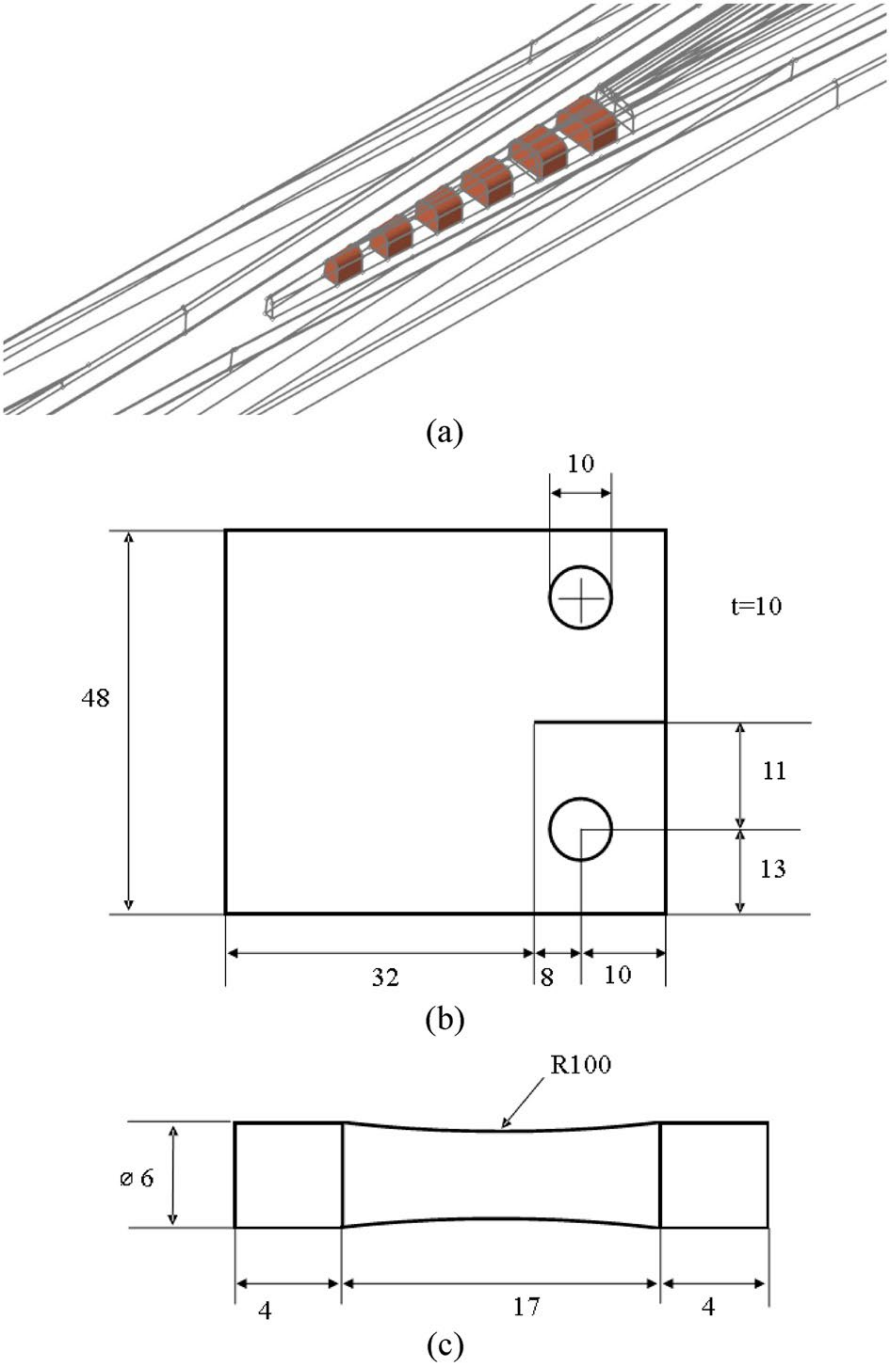
Geometries used in this study.

In the current study, three different base geometries are utilized with different numerical configurations to simulate several cases (i.e. comparison between FEM and PD, verification of crack on midplane, comparison between linear and adaptive time-mappings, validation of crack initiation part, validation of crack propagation part and fatigue simulations of a crossing nose). Those three geometries are a CAD geometry of a turnout provided by a local manufacturer in Turkey (Fig. 2a), a standard CT specimen (Fig. 2b) and a fatigue test specimen (Fig. 2c) obtained from^58^. Here, it should be emphasized that the fatigue specimen used in the current study is simplified version of a standard fatigue test specimen. It is a middle section of a standard specimen where the failure occurs. It is modified by a radius of 100 mm, which results in 10% reduction of the diameter, to facilitate failure at the centre of the specimen. Otherwise, it was observed that failure takes place close to the loading points due to the same loading conditions along the specimen. Another rationale behind this approach is the fact that the fatigue simulation of PD model is strain-controlled and the modification does not influence the stiffness of PD bonds. The impact of the modification on force equilibrium was found to be limited after a comparison between the modified model and the base model. The maximum discrepancy was below 0.4% in terms of displacements.

Another remark is about CT specimen used in the crack propagation simulation of the model itself having material properties of titanium alloy. In that case, the geometry in Fig. 2b is scaled down by 0.625. All three geometries are presented in Fig. 2.

The material model used throughout all simulations is a linear-elastic state-based PD material model, properties of which are decided with reference to a common R260 grade rail steel, 7075-T651 aluminium alloy and Ti6Al4V titanium alloy. Material properties and fatigue parameters are extracted from^59–62^ and presented in Table 1. Mesh properties for each geometry differ from each other owing to geometric challenges encountered during discretization. It is apparent that discretization of CT specimen is a trivial task as it is a single block with a constant cross section. Therefore, it is discretized with structured points. The distance between each node is specified as 0.625 mm and 1 mm for titanium alloy and rail steel models, respectively. But meanwhile, 2 mm discretized geometry is used for the comparison of time-mappings. On the other hand, turnout geometry and fatigue specimen are generally discretized with unstructured nodes due to their topology. Therefore, the distance between each point varies within the geometries. Average distances are around 1 mm for turnout geometry and 0.5 mm for fatigue specimens. The horizon parameter of $3{\Delta }x$ is selected for each case as recommended in^33^.

**Table 1:** Material properties and fatigue parameters.

	Rail	Aluminum	Titanium	Units
Density	7850	2810	4420	${\text{kg}}/{\text{m}}^{3}$
Modulus of elasticity	207	71.7	110	${\text{GPa}}$
Poisson’s ratio	0.295	0.306	0.333	
A1	505	14,514	505	
A2	17,500	17,500	1.7e11	
m1	3.01	3.472	3.01	
m2	2.8756	2.8756	4.757	
$\tau$	0.01	0.01	0.01	

A standard 49E1.1-9 R300 turnout geometry is cut by 40 mm sections as shown in Fig. 2a. Each section also has a distance of 40 mm apart from each other. The length of each section is two times longer than the calculated contact patch length, which is sufficient according to^46^. In total, 440 mm length of a crossing nose is considered in terms of potential contact. The total length is generously decided to include all potential contact cases in comparison to field findings^63,64^. Contact area parameters are separately calculated in accordance with Hertzian Elastic Contact Theory and applied as a boundary condition in PD simulation. It is noteworthy that the available contact theories in PD are unfortunately insufficient to obtain a contact patch as they specifically involve point-to-point contact. In other words, surface-to-surface contact in FEM is not available in PD and therefore, contact is an open research topic in PD applications. As far as the other conditions are concerned, two more boundary conditions are applied to the geometry. First, each section has been fixed in all directions at the bottom layer with $\delta$ length. Secondly, the side walls of each section, where the geometry was cut by planes, are fixed in longitudinal and lateral directions. And finally, the boundary conditions for other geometries are set as follows. For CT and fatigue geometries, the displacements in lateral directions are restricted. Likewise, the loads are applied from the ends of the geometries in vertical directions. The loading points are neglected in damage calculation to provide constant loads and prevent “PD force singularity”. Loads are distributed over all points within the horizon due to the non-locality of PD^65^.

**Figure 3:**
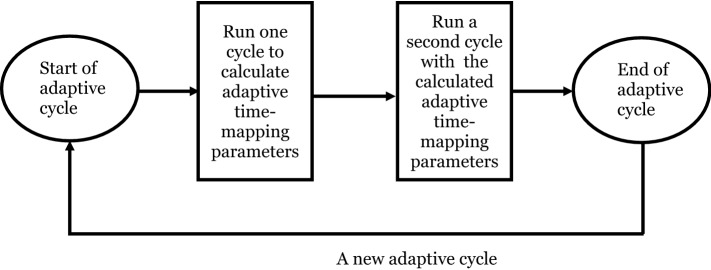
An illustration of adaptive time-mapping procedure.

The workflow of the proposed adaptive time-mapping is illustrated in Fig. 3. It is apparent that the adaptive time-mapping code can be modified further. For instance, PD solvers can be altered to go back to the previous state after the calculation of adaptive parameters. It is noteworthy that “going back to previous state” would remove the necessity of “sensitivity constant”. Nevertheless, it requires an extensive validation process since it is a modification of the solver system. Therefore, the current methodology producing some errors is assumed to be sufficient for the current study.

## Results and discussions

In this section, the results of fatigue simulations are presented. An open-source software, “Peridigm”^66^, is modified in order to implement the methods above and used in the current study. Due to high computational costs, the simulations are conducted on SULIS and BEAR HPC systems. The reason behind signing-up two different HPC systems is because of the limits of allocated resources. Both systems have cutting-edge technology and show similar performance. Each simulation in this study consumes a different amount of resources from the dedicated budget. The maximum computational cost of a single simulation was 60 × 6 CPU cores × days.

### FEM-PD verification

**Figure 4:**
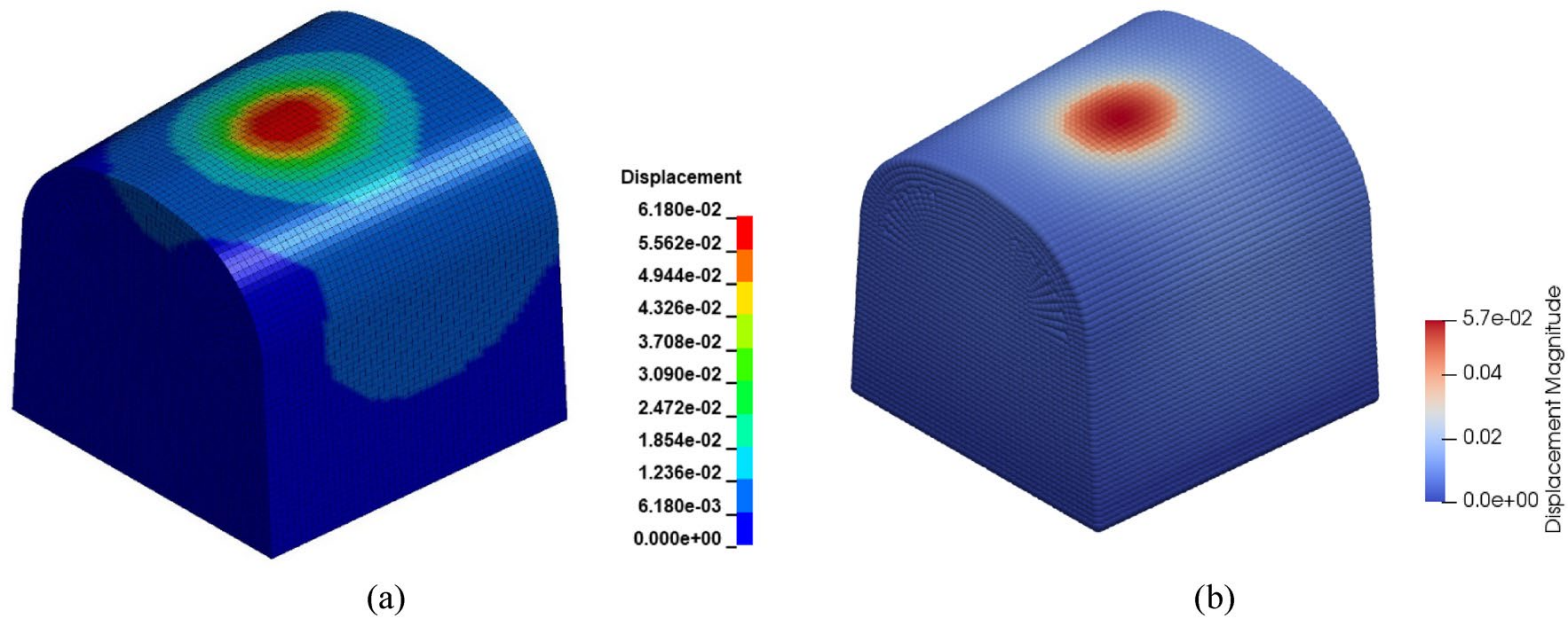
Numerical geometries, (**a**) FEM (**b**) PD. The magnitudes are in mm.

A numerical model entailing a contact patch that occurs between rail and wheel, cannot be validated by experiments since it is unlikely to measure contact parameters in the field. Therefore, PD model of a crossing nose, details of which is given previously, has been verified by the well-known FEM. The models are illustrated in Fig. 4. To verify PD model, six different scenarios are tested in both environments. Then, the models are compared in terms of displacements. It is noteworthy that PD theory basically does not retain stress terms. In fact, the same argument is also valid for FEM. In the theory of FEM, the main focus is fundamentally made on the displacements. The stress terms are calculated later in the so-called post-processing part. Hence, the validation by displacements is regarded as a rational approach.

**Figure 5:**
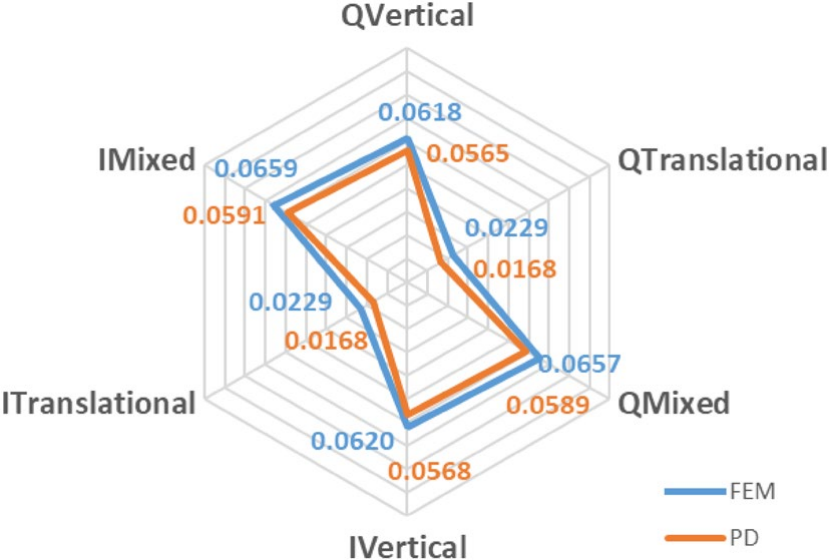
Comparison between FEM and PD in terms of maximum displacements in mm.

In Fig. 5, a comparison between PD and FEM models is presented in terms of the maximum displacement. In the figure, the letter “Q” and “I” represent “Quasi-static loading” and “Impact loading”, respectively. Likewise, “Vertical”, “Translational” and “Mixed” terms show the loading directions. Here, the translational direction is the rolling direction of the wheel and the mixed loading is the combination of vertical and translational loadings. As can be seen from the figure, both models estimate similar displacements, ranging from 1.7e − 2 mm to 6.2e − 2 mm. It is clear that PD model exhibits a conservative form and slightly underestimates the displacements in all scenarios. The discrepancies between two methods in all cases are close to each other in terms of magnitude, as the upper and lower bounds are 5.0e − 3 mm and 6.0e − 3 mm, respectively. In other words, there is a consistency in the error, which means that the error characteristics is related to PD theory itself. A likely source of the error is so-called “surface effects” in PD, which arise from insufficient bonds for the points close to boundary surfaces. The surface effects cause softer material behaviours at locations close to boundaries. To mitigate surface effects, the basic treatment is to adjust the stiffness of the bonds close to surfaces. However, a solution brought to the surface effects cannot eliminate the errors completely. As the focal point in the current study is the contact patch that lays on the surface of the rail, the errors are always unavoidable at this point. Here, it should be emphasized that the treatment of surface effects is an open research area and is ignored within the scope of this study, In conclusion, PD model of the crossing nose is accepted as accurate with a degree of confidence with reference to FEM.

### Verification of crack on mid-plane

The novel “crack on mid-plane” method previously developed by authors^49^ is also verified with the experimental data in^60^ in this study. The method is crucial to track crack formation and propagation since the commonly used method of “nodal damage value” in Eq. (32) produces different outcomes for different nodal damage values. In more details, Eq. (32) describes that the damage for a point is the ratio of total broken bonds over total non-broken bonds. Since the equation does not exclude any broken bonds irrelevant to crack propagation, it produces several outcomes depending on the magnitude of the nodal damage value.

**Figure 6:**
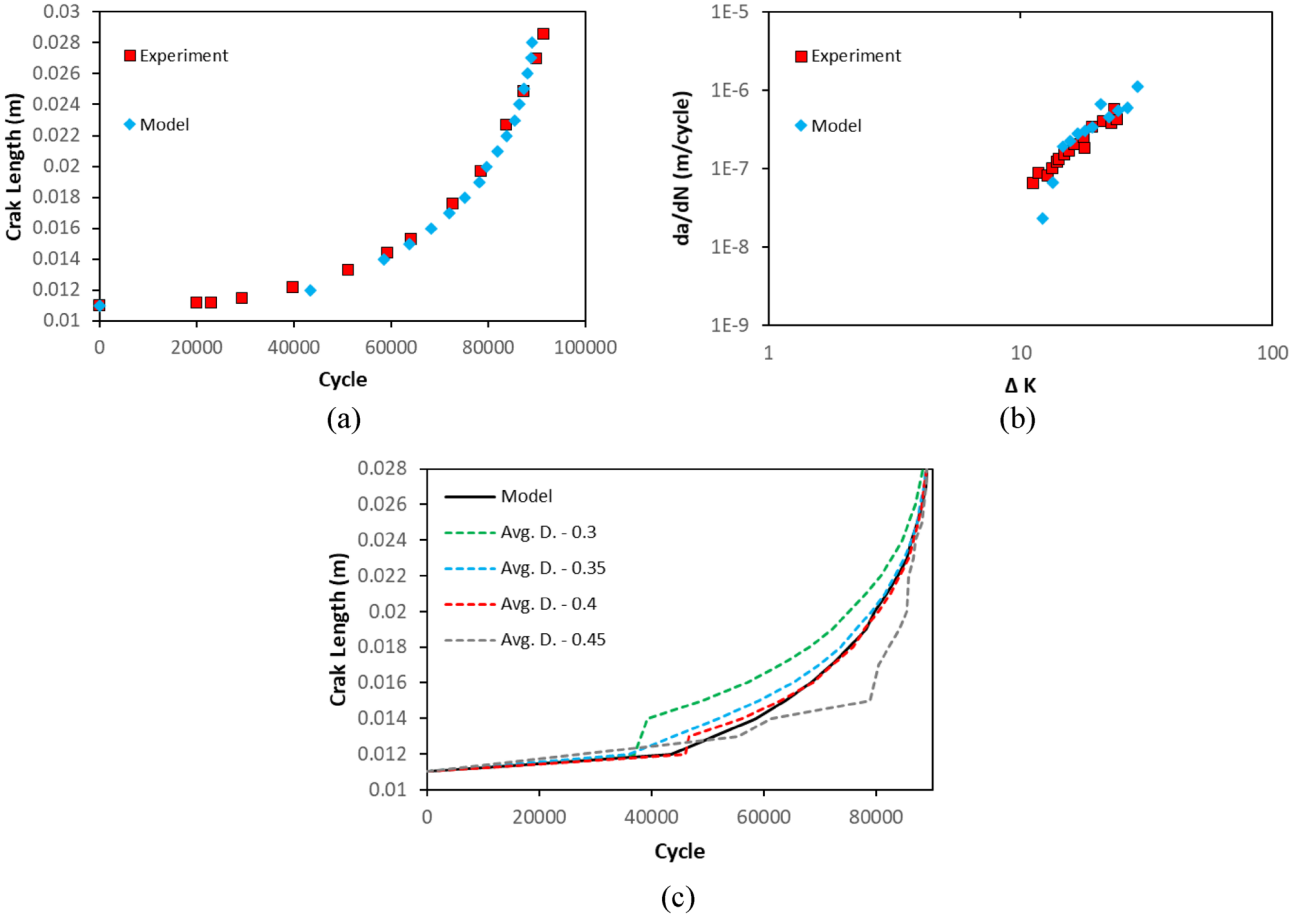
(**a**) A comparison between experimental data and the method in terms of crack length vs cycles. (**b**) A comparison between experimental data and the method in terms of crack propagation speed vs stress intensity factor. (**c**) A comparison between different nodal damage values vs the method in terms of crack length vs cycles.

**Figure 7:**
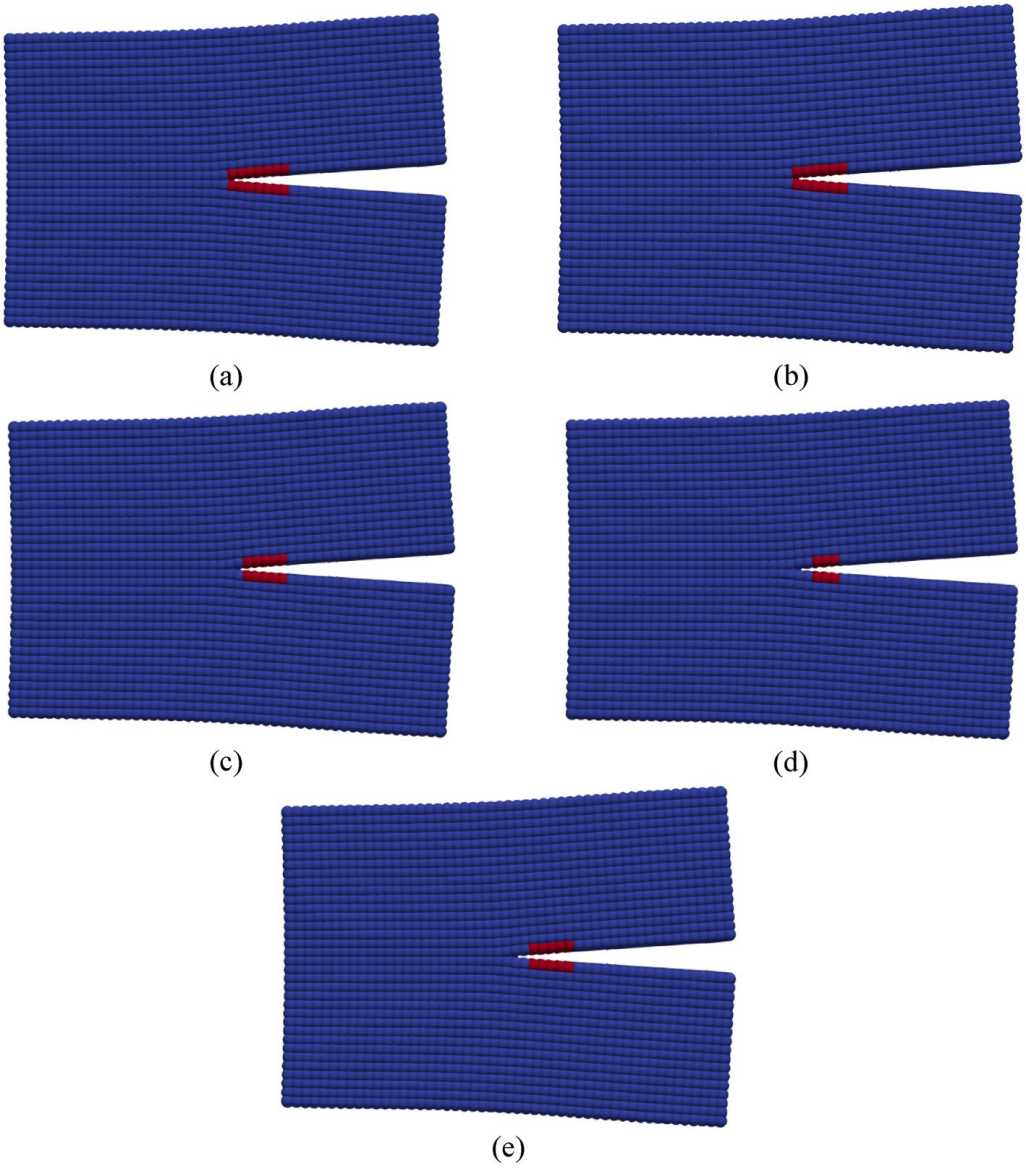
Crack propagation at 69.8 k cycles when (**a**) nodal damage value of 0.3, (**b**) nodal damage value of 0.35, (**c**) nodal damage value of 0.4, (**d**) nodal damage value of 0.45, (**e**) the crack on mid-plane method. Displacements are magnified by 50x.

A CT specimen in Fig. 2b, assigned with rail material, is used in the simulations that utilize linear time-mappings for cycle calculations. In Fig. 6a, b, a comparison between experimental data and “crack on mid-plane” is illustrated. With reference to the data in Fig. 6b, it has been statistically observed that the mean errors of the model and the experiment with reference to the regression line of the experimental data at logaritmic scale are 1.58 and 1.28, respectively. In addition, the standard deviations of the errors of the model and the experiment are 3.58 and 0.9, respectively. Both figures confirm that the method is able to mimic the crack propagation behaviour of the CT specimen and therefore, is suitable to use in PD simulations. The advantage of the method can also be seen in Fig. 6c, where different nodal damage values and the method of “crack on mid-plane” are compared. It is clear in the figure, various damage values produce different crack behaviours. The impact of different nodal damage values is also visualized by 50 × magnified pictures in Fig. 7. The length of the crack estimated by various nodal damage values varies substantially, ranging from 14 to 18 mm after 69.8 k cycles, whereas the method produces a single outcome giving 16 mm crack length.

32 $$\phi \left( {x_{i} } \right) = 1 - \frac{{\mathop \smallint \nolimits_{{H_{x} }}^{ } w\left( {x_{ij} } \right) \cdot {\text{d}}V_{{x_{j} }} }}{{\mathop \smallint \nolimits_{{H_{x} }}^{ } {\text{d}}V_{{x_{j} }} }}$$

### A comparison between adaptive time-mapping and linear time mapping

The method of ‘adaptive time-mapping’ is compared with no time-mappings as well as linear time-mappings proposed in the reference study. As aforementioned, the point of employing linear time-mapping is to reduce the simulation process time with an acceptable compromised error level. A CT specimen with 2 mm meshing is used to reduce the simulation time. Likewise, the fatigue constants are adjusted, since the accuracy of the simulation may be ignored at this point. In other words, a CT specimen with random fatigue parameters providing a lower simulation time is used to test time-mapping configurations. The idea behind using a random specimen is to avoid unnecessary and impractical simulation processes on HPC by finding an optimum time-mapping configuration that yields a shorter simulation time with an acceptable error rate. It can be considered as test runs for different time-mapping configurations under the same numerical configurations. Referring to a real sample (i.e. a steel specimen model having 100 k cycles life), it would be computationally expensive simulation in which no time-mapping is applied (as shown in Table 2). Note that the validation of the models with a selected time-mapping configuration will be demonstrated in the following sections.

**Table 2:** Comparison between different time-mapping scenarios.

	R	L.1	L.01	L.001	A.0	A.1	A.01	A.05	A.001	H.05.01
Error (%)	0	1.15	9.13	61.7	34.6	− 3	5.6	1.85	11.1	7.85
Cost (s)	60,000	5700	580	66	960	430	1000	520	1200	320

Table 2 depicts the relative errors for a complete failure of the specimen and computational cost for 2 mm crack propagation under different time-mapping configurations. The specimen in the reference case, as represented by the letter R in the table, has no time-mapping and fails after 19.8 k cycles. The other letters L, A, H represent linear, adaptive and hybrid (adaptive + linear) time-mappings, respectively. The numbers give decimal parts of linear time-mapping constants or sensitivity constants of adaptive time-mapping. For instance, L.01 means “Linear time-mapping with a constant of 0.01”. For hybrid time-mapping, the first part of the number gives sensitivity constants and the second part provides linear time-mapping constants. By looking at the table, it becomes clear that adaptive time-mapping shows significant performance in comparison to no time-mapping. Nevertheless, the pure application of adaptive time-mapping presents a significant error rate originated from the characteristic structure of code. The error can be minimized by tuning the sensitivity constant. For this purpose, several sensitivity factors are tested. As shown in the table, the relative errors are − 3%, 1.85%, 5.6% and 11.1% for sensitivity constants of 0.1, 0.05, 0.01 and 0.001, respectively. It is obvious that the performance of an adaptive time-mapping with a 0.05 sensitivity outperforms all other constants as well linear-time mapping with 0.01 and 0.001. The sensitivity constant can be adjusted further, however, in this study, such action is avoided for the number of reasons. First, it is not feasible to consume more HPC resources on tuning the constant. Secondly, the sensitivity is applied to compensate the error resulting from the code structure. Hence, it is believed that an optimization of the adaptive time-mapping code would remove the necessity of sensitivity. At this point, the performance of the code is sufficient and no optimization is need within the scope of the current study. Finally, the linear time-mapping constant of 0.001 presents the largest error, showing that a selection of linear time-mapping constant must be done carefully.

It is apparent in the table that the adaptive time-mapping is significantly efficient in terms of simulation time when compared to no time-mapping case or linear time-mapping constants such as 0.1. In the table, it is also visible that the lower linear time-mapping constants also reduce the simulation time. Nonetheless, there is a reverse correlation between a linear time-mapping constant and the error rates. The lower the linear mapping constant is, the much higher is the error rate.

As explained in the methodology, a hybrid approach (a combination of adaptive and linear time-mappings) further increase the efficiency due to the characteristic structure of the code. With reference to Table 2, H.05.01 seems to be an optimal selection in terms of error and simulation time. Here, it is crucial to emphasize that the length of the simulations in Table 2 are adjusted to a lower cycle failure scenario to be able to run a reference simulation without a time-mapping. Otherwise, the computational cost of the simulation with no time-mapping would be extremely high.

To present more evidence about the effectiveness of adaptive time-mapping, H.05.01 is compared to L.01 for two more cases such as low and high cycle failures. The outcomes of the comparison for 2 mm crack propagation are presented in Table 3 as it provides a concise picture where H.05.01 becomes superior over L.01 by an 88.6% reduction in the computational cost in the case of a high cycle failure without any significant loss in the estimation. In conclusion, a time-mapping configuration is a necessity to run a fatigue simulation in a PD environment and a trade-off must be considered between the error rate and the cost. With reference to the above findings, the hybrid approach performs notably. Therefore, H.05.01 time-mapping configuration is selected and applied in the current study in general.

**Table 3:** Comparison between long, short and reference time-mapping configurations.

	Cycles to 2 mm crack			Cost		
	Case I	Case II	Case III	Case I	Case II	Case III
H.05.01	4114	16,047	163,446	130	320	760
L.01	4300	16,300	166,600	170	580	6700
Difference (%)	4.3	1.5	1.8	23.5	44.8	88.6

### Validation of crack initiation

**Figure 8:**
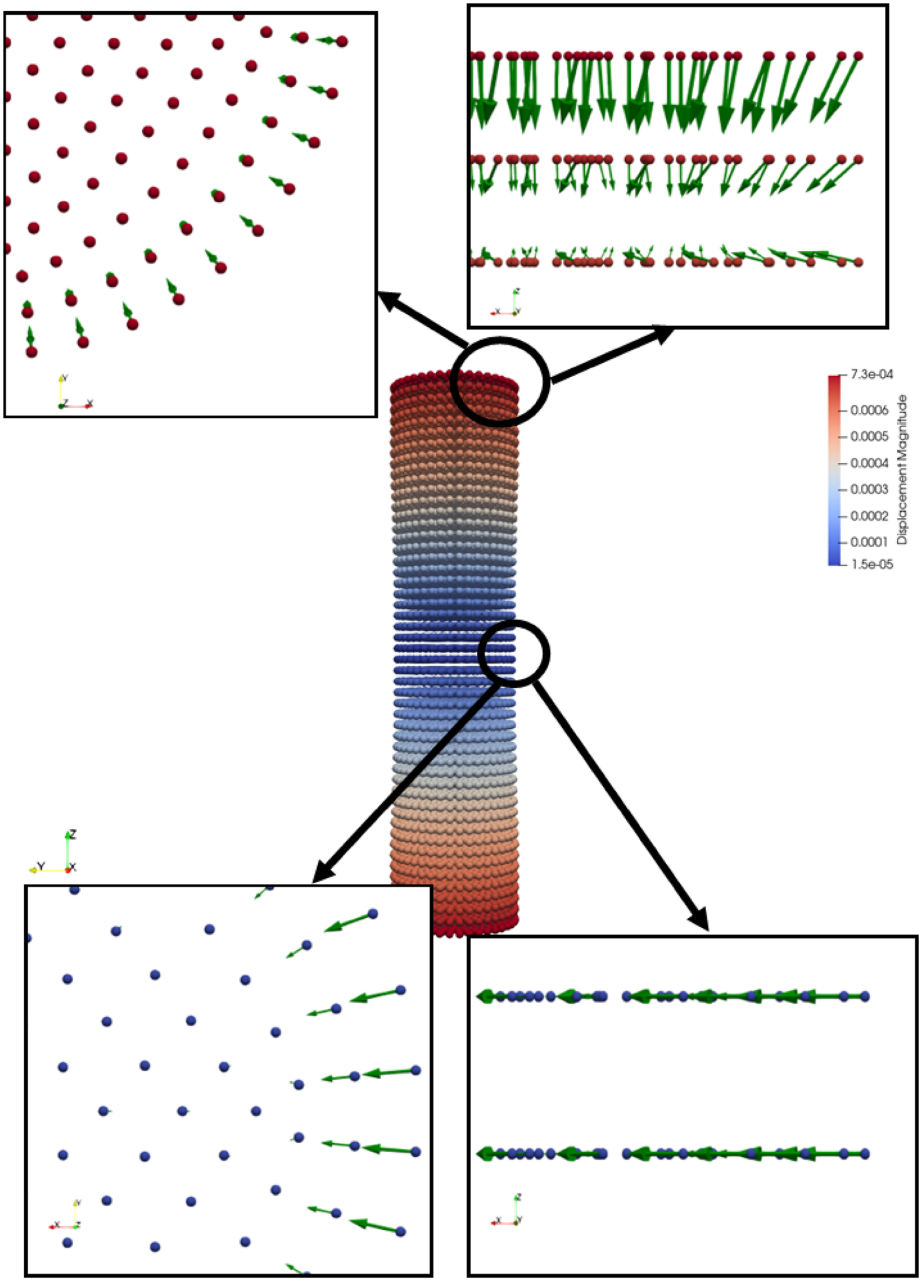
Force density vectors (resultant force per unit volume) at the maximum displacement for fatigue specimen.

To employ the fatigue model to evaluate fatigue damage over a crossing nose, each stage of the fatigue model is validated against experimental findings. In this section, the crack initiation part is validated against the two studies in^59,61^ that present the correlation between strain and life for an ordinary rail and aluminium alloy. As mentioned in the methodology earlier, the rationale behind referring to strain-life data is because of the characteristic of PD which does not include the concept of stress implicitly. In Fig. 8, force density vectors at the maximum displacement are illustrated for cut sections at middle and top parts of the specimen. Force density vectors are resultant force vectors per unit volume. As shown in Fig. 8, the symmetry conditions at the middle part cause lateral forces that compress the specimen under tension in the lateral direction. This behaviour is the Poisson’s effect. On the other hand, the resultant forces at the top part also have vertical component due to external forces. The external force is applied in terms of the prescribed displacement which provides the strain controlled experiment. Here, it should be emphasized that force density vectors do not provide any information about the individual bond behaviours.

#### Rail steel

**Figure 9:**
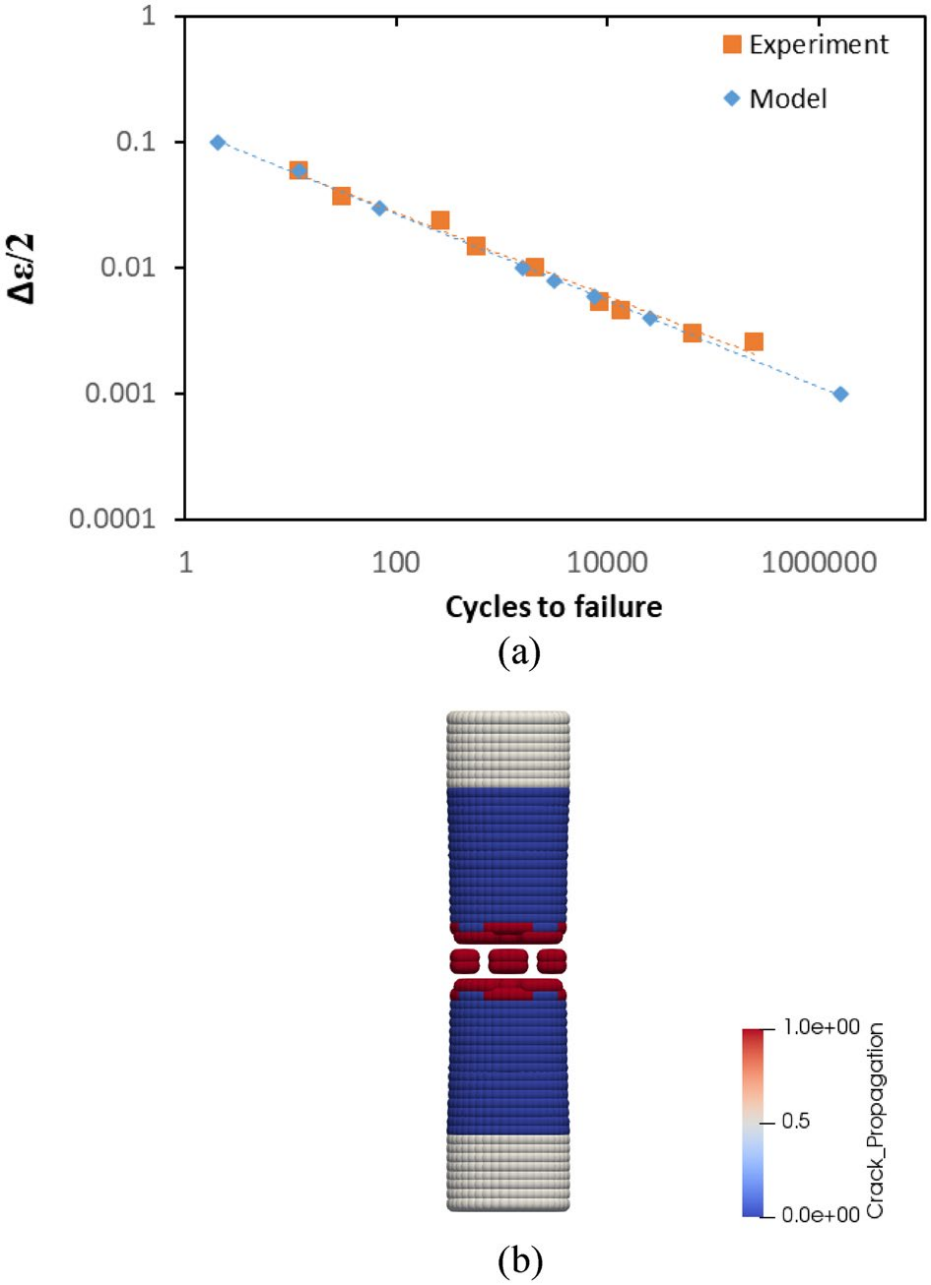
Rail steel. (**a**) A comparison between the experiment and PD simulation in the case of crack initiation. (**b**) A picture of failure mode for crack initiation from PD simulation.

PD fatigue model estimates strain-life behaviour of an ordinary rail close to the experimental data as shown in Fig. 9a. For instance, the fatigue life of rail specimens is lower than 100 cycles if the strains are above 1%. Statistically, the mean error of the model with reference to the regression line of the experimental data at logaritmic scale is 0.938 whereas the mean error is 0.35 for the experiment. The standard deviations of the errors of the model and experiment are 1.58 and 2.78, respectively. Overall, PD model presents a similar characteristic with experimental data, which is known as strain-life curve. One may be questioning the visible discrepancy that happens at a strain level of 0.001 which is normally below the fatigue limit. As theoretically the life of the material goes to infinity below the fatigue limit, it is unlikely to simulate a scenario that goes to infinity. Therefore, the fatigue limit is deliberately discarded in the simulation to show the correlation between the number of cycles to failure and lower strain values. Finally, in Fig. 9b, the failure mode of the specimen with a strain value of 0.1 is given as a representative example.

#### Aluminium alloy

**Figure 10:**
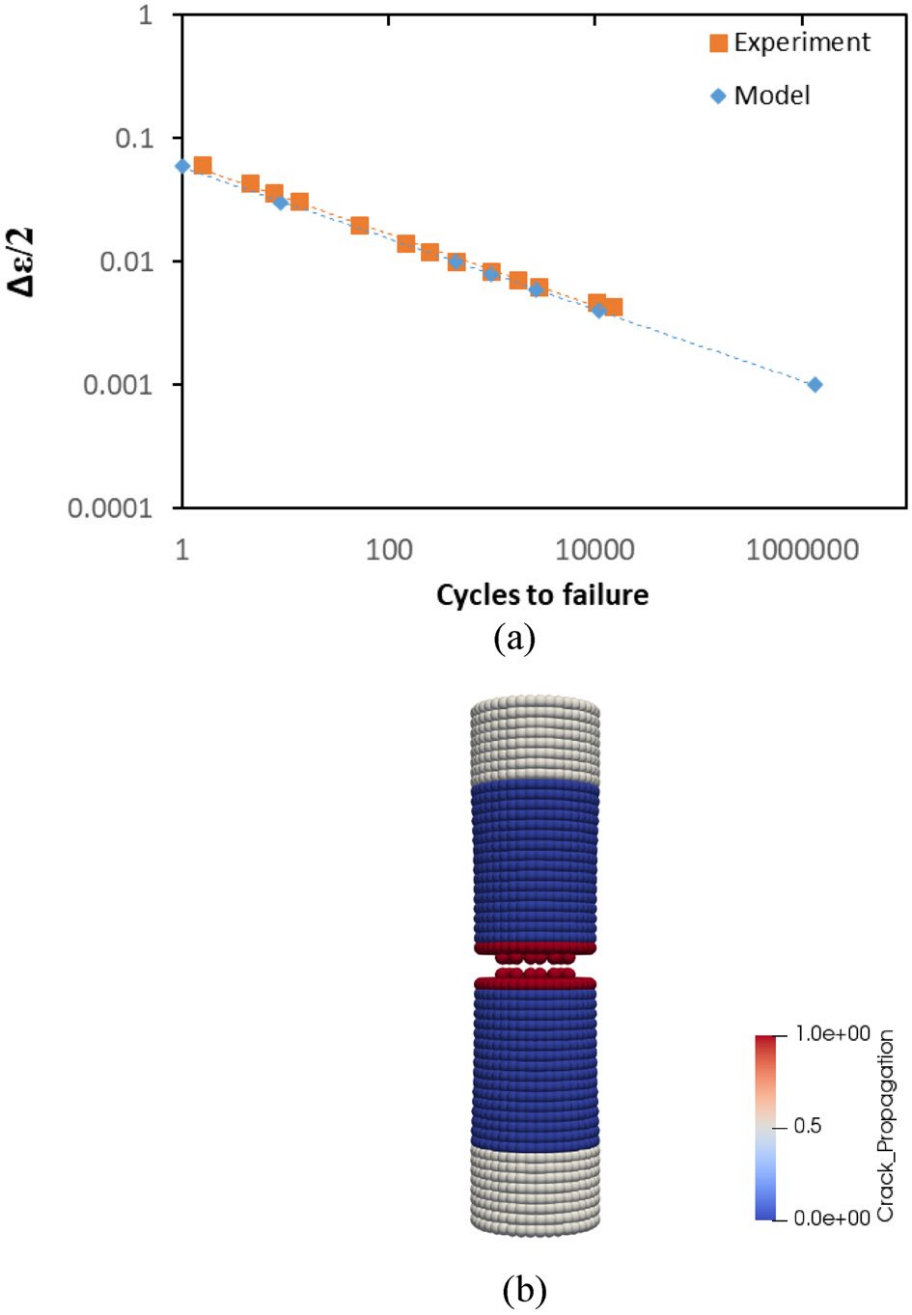
Aluminium alloy. (**a**) A comparison between the experiment and PD simulation in the case of crack initiation. (**b**) A picture of failure mode for crack initiation from PD simulation.

Similar to rail steel, PD model also accurately reflects the fatigue behaviour of aluminium alloy. In Fig. 10a, data points from the experiment and model agree with each other. Furthermore, the gradients of trend lines of those data points are similar. The mean errors of the model and the experiment with reference to the regression line of the experimental data at logaritmic scale are 1.86 and 0.04, respectively. The standard deviation of the model is 2.18 whereas it is 1.41 for the experiment. In Fig. 10b, the failure mode of the specimen is provided for a strain value of 0.06 as an example. Similar to rail steel, failure occurs at the centre. In all cases, failure mode happens similarly.

In conclusion, with reference to Figs. 9 and 10, it can be concluded that the initiation part of the fatigue model that employs “crack on mid-plane” and the H.05.01 “adaptive time-mapping” configuration sufficiently mimics the experimental behaviours. Consequently, it can be assumed as valid to be used in PD simulations.

### Verification of crack propagation

The second part of the fatigue model involves the crack propagation stage. This stage is validated against experimental data presented in^60,62^ for rail steel and titanium alloy, which were loaded with 14 kN (R = 0.2) and 7 kN (R = 0.1), respectively. For the validation, a CT specimen, the details of which are given in the methodology, is utilized.

**Figure 11:**
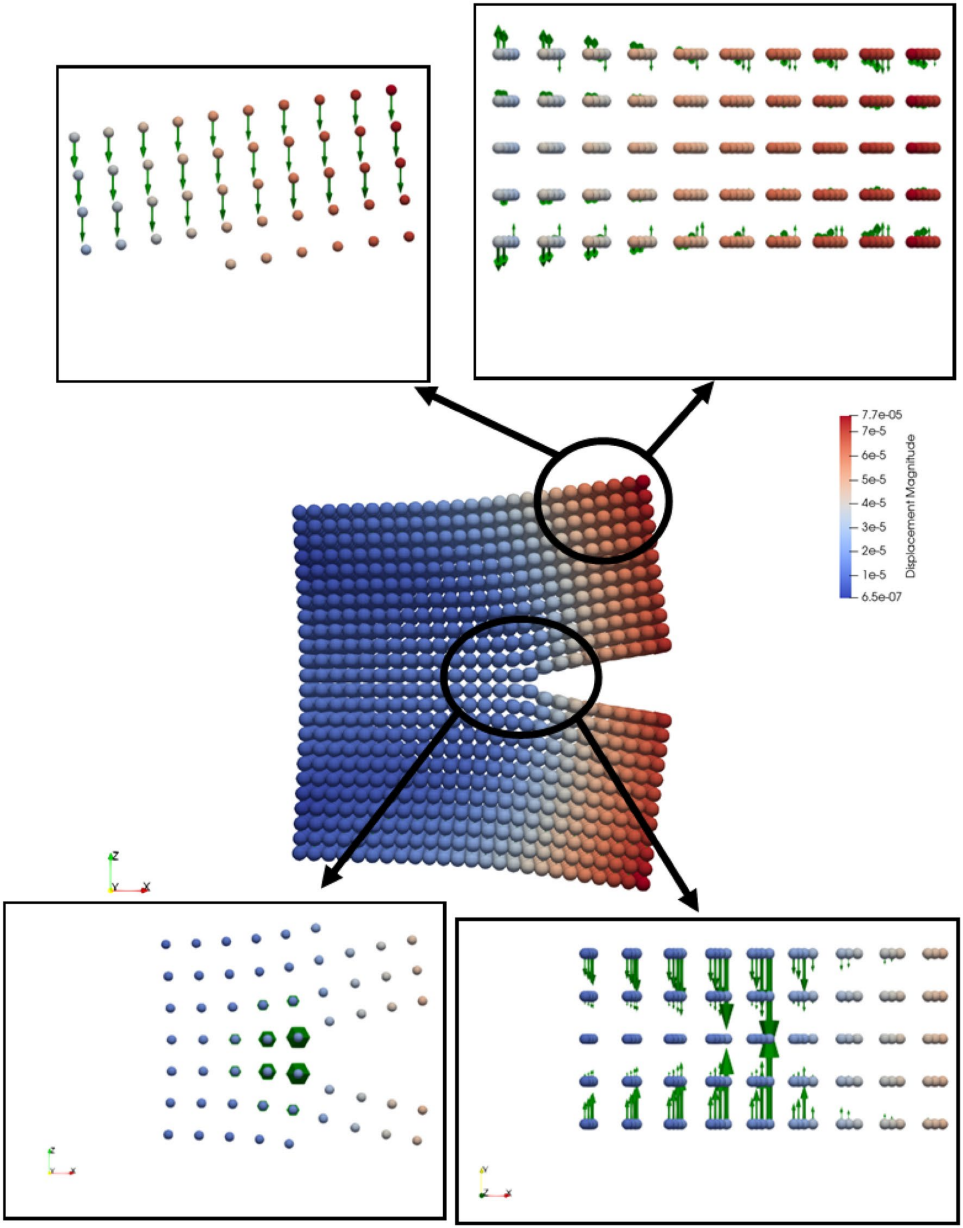
Force density vectors (resultant force per unit volume) at the maximum displacement for SEN speciment.

Figure 11 demonstrates the force density vectors for the middle and top sections of the SEN specimen at the maximum displacement. Similar to the force density vectors of the crack initiation part, the symmetry conditions at the middle part present lateral forces compressing the specimen. Moreover, the resultant forces at the top part also have relatively large vertical and small lateral components due to external forces which are applied as body forces over three layers.

#### Rail steel

**Figure 12:**
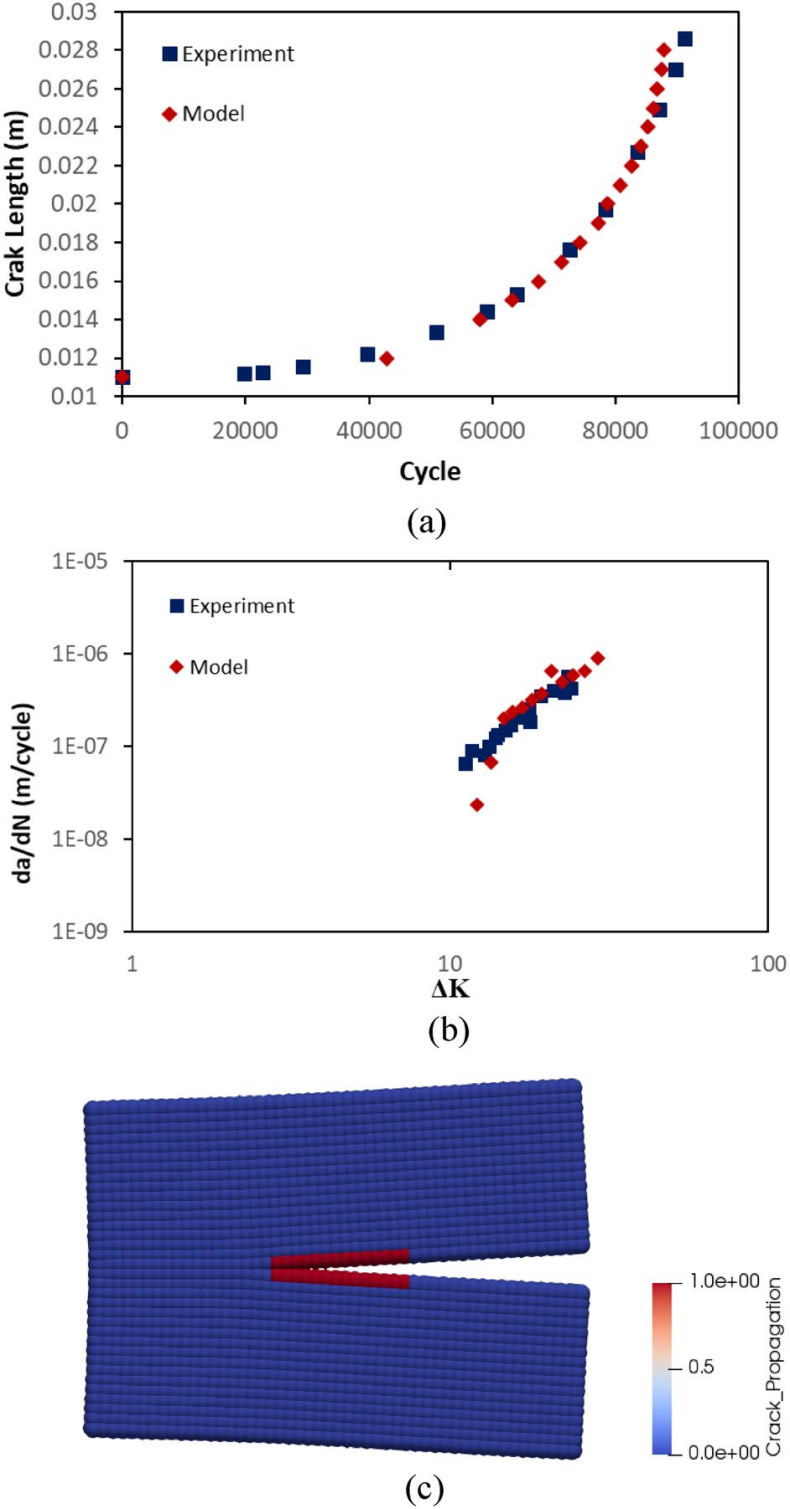
Results for rail steel model. (**a**) A comparison between the experiment and PD simulation in terms of crack length vs cycle. (**b**) A comparison between the experiment and PD simulation in terms of crack growth speed vs stress intensity factor. (**c**) A picture of crack propagation after 83 k cycles from PD simulation.

A comparison between PD model and experimental data is depicted in terms of crack propagation for rail steel in Fig. 12a. As can be seen from the figure, the fatigue growth curves for the experiment and the model follow a similar path and the failures happens after 85 k cycles. The model fails slightly earlier than the experiment. In PD model, the constant mesh size and the concept of bonds allow to measure the crack propagation speed when the crack propagates from one point to another. In other words, the measurement of the crack propagation speed in PD is dependent on the mesh size. That is particularly observable at lower cycles, where the data points are less dense in the model. In Fig. 12b, the correlation between stress intensity factor and crack propagation speed is also shown. In general, the linearity of experimental and model data is similar. However, the crack propagation speed for the model is lower than the experiment for lower stress intensity factors. It result from the calculation of stress intensity factor which ignores the non-locality and mesh dependency of PD crack behaviour. The statistical assesment of the data in Fig. 12b shows that the mean errors of the model and the experiment with reference to the regression line of the experimental data at logaritmic scale are 1.76 and 1.28, respectively. In addition, the standard deviations of the errors of the model and the experiment are 3.11 and 0.9, respectively. The reconciliation level between the model and experiment might be increased by employing finer meshes but its cost then exponentially increases. In Fig. 12c, a picture of crack propagation at 83 k cycles is also presented.

#### Titanium alloy

**Figure 13:**
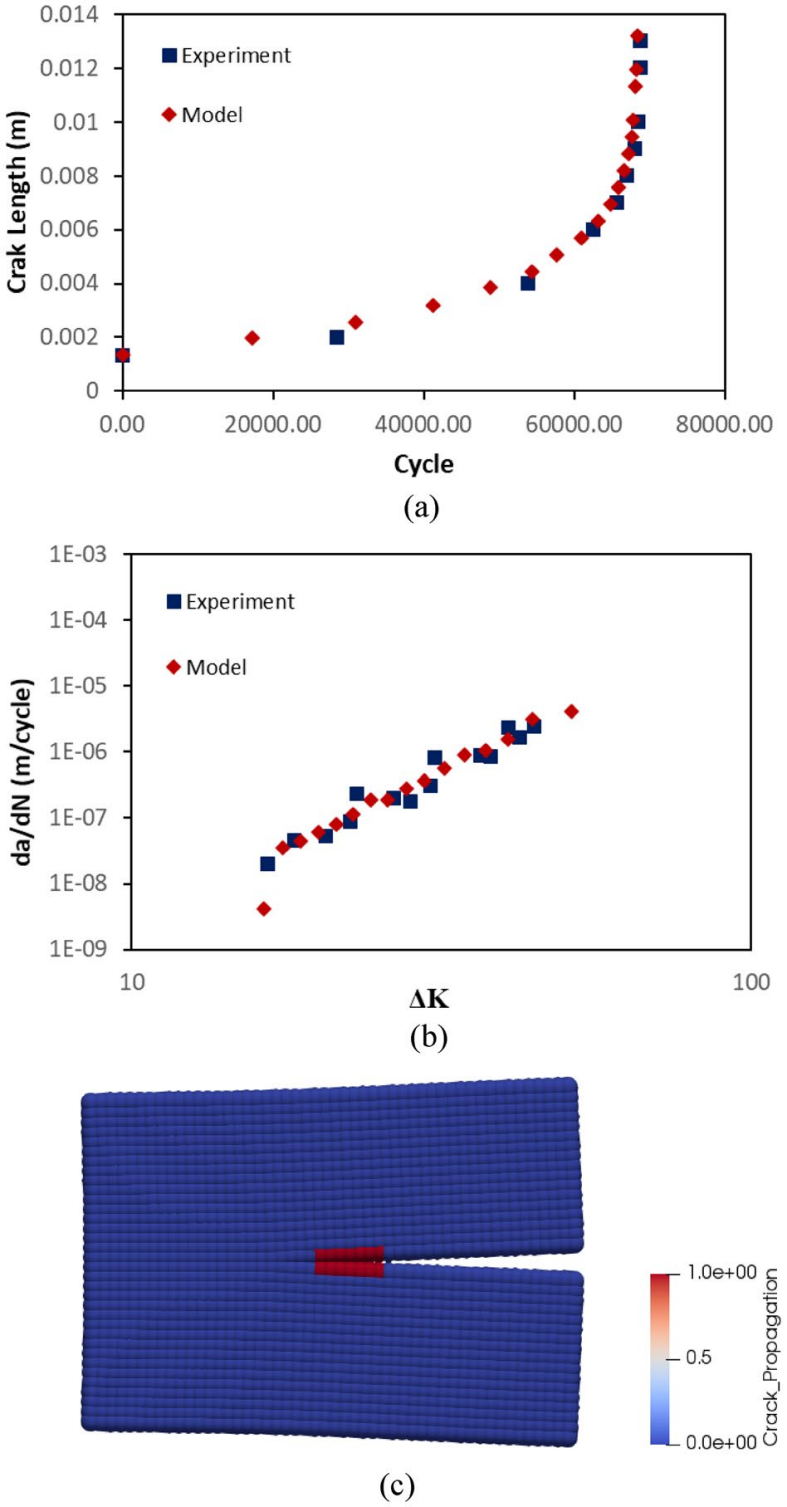
Results for titanium alloy model. (**a**) A comparison between the experiment and PD simulation in terms of crack length vs cycle. (**b**) A comparison between the experiment and PD simulation in terms of crack growth speed vs stress intensity factor. (**c**) A picture of crack propagation after 60 k cycles from PD simulation.

The crack propagation behaviour of titanium alloy PD model also coincide with the experimental findings. In Fig. 13a, the crack lengths at different cycles are presented. The failure occurs after 65 k cycles in both model and experiment. In Fig. 13b, crack propagation speed of titanium alloy model is compared with the experimental data in terms of stress intensity factor. Following the statistical assesment of the data in Fig. 13b, it has been found that the mean errors of the model and the experiment with reference to the regression line of the experimental data at logaritmic scale are 0.68 and 5.08 while the standard deviations are 2.63 and 4.85, respectively.The confirmation level between PD model and experiment is considerably high. In Fig. 13c, a representative picture is given for crack propagation at 60 k cycles.

Despite discrepancies at lower and higher cycles, the crack propagation behaviour of PD models for rail steel and titanium steel is still in line with the general view on the application of Paris law. As well-known, Paris law is limited to the stable crack propagation region. In other words, it is inefficient at lower and higher cycles. In general, PD fatigue model enriched with “adaptive time-mapping” and “crack on mid-plane” is capable of reflecting the nature of crack propagation in a CT specimen. Therefore, the propagation part of the fatigue model can also be assumed as valid in order to use in fatigue simulations of a crossing nose.

### Life estimations for crossing nose sections

Following the validation of each stage of the fatigue model, six different cross-sections of a crossing nose as previously mentioned in the methodology are numerically tested in PD environment in order to calculate the total life of each cross-section. The numbering of each cross-section is done with reference to the position of nose, where the first cross-section is the closest one to the nose. Each cross-section has different geometry and a contact patch that is specific to that geometry. Contact patches are positioned at the centre point of the rail surfaces. Load magnitudes and frequencies are obtained from the authors’ previous study^2^ and applied as impact forces with a magnitude of 130 kN and a frequency of 200 Hz over contact patches.

**Figure 14:**
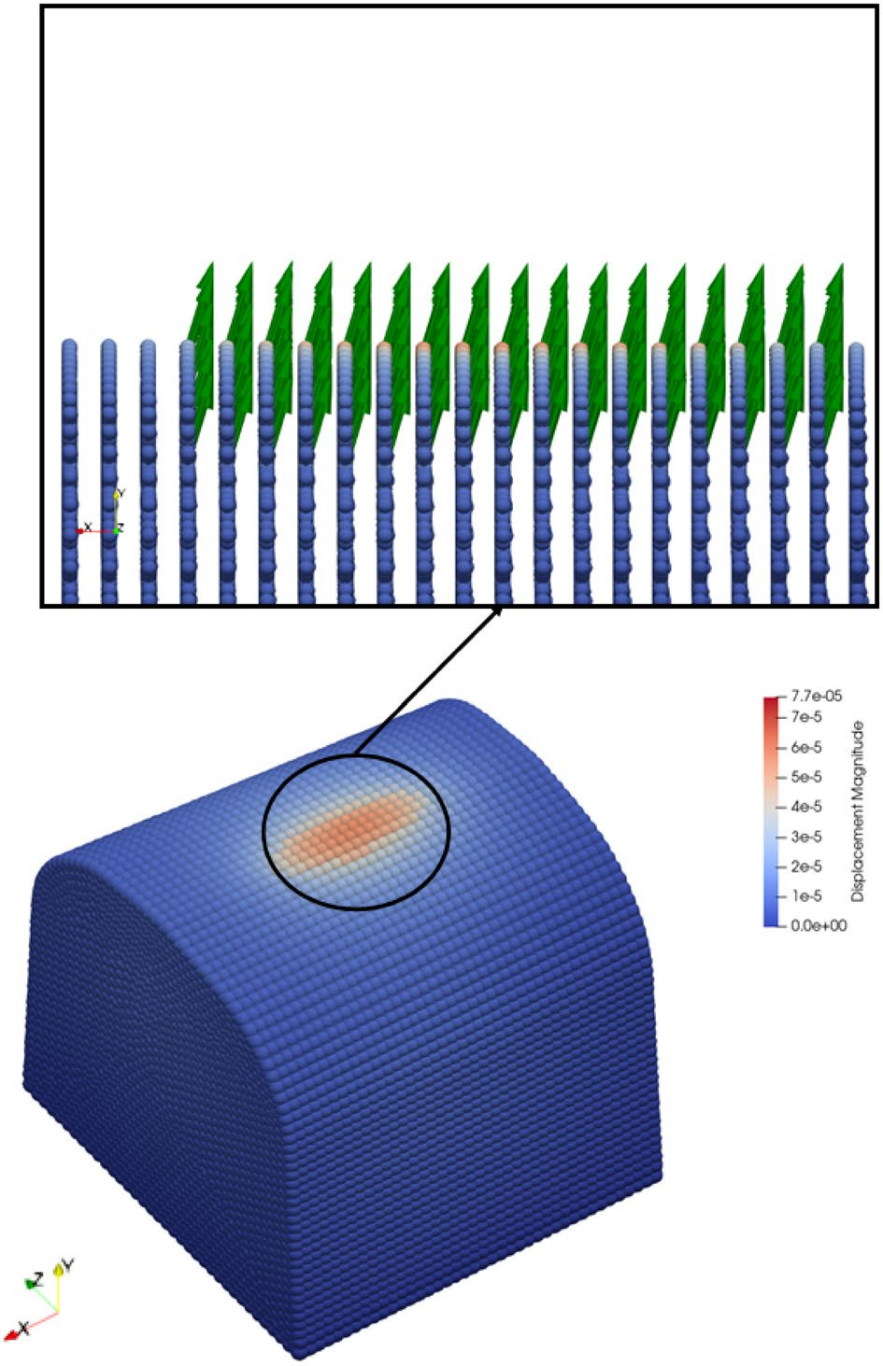
Force density vectors (resultant force per unit volume) at the maximum displacement over a crossing nose.

Figure 14 illustrates the force density vectors over a crossing section. As clearly shown in the figure, the force density vectors are a response to external contact forces. Here, it is worth mentioning that other nodes also have force density vectors but the magnitude of those vectors are comparably lower than the ones on the contact patch. That is why they are imperceptible in the figure.

**Figure 15:**
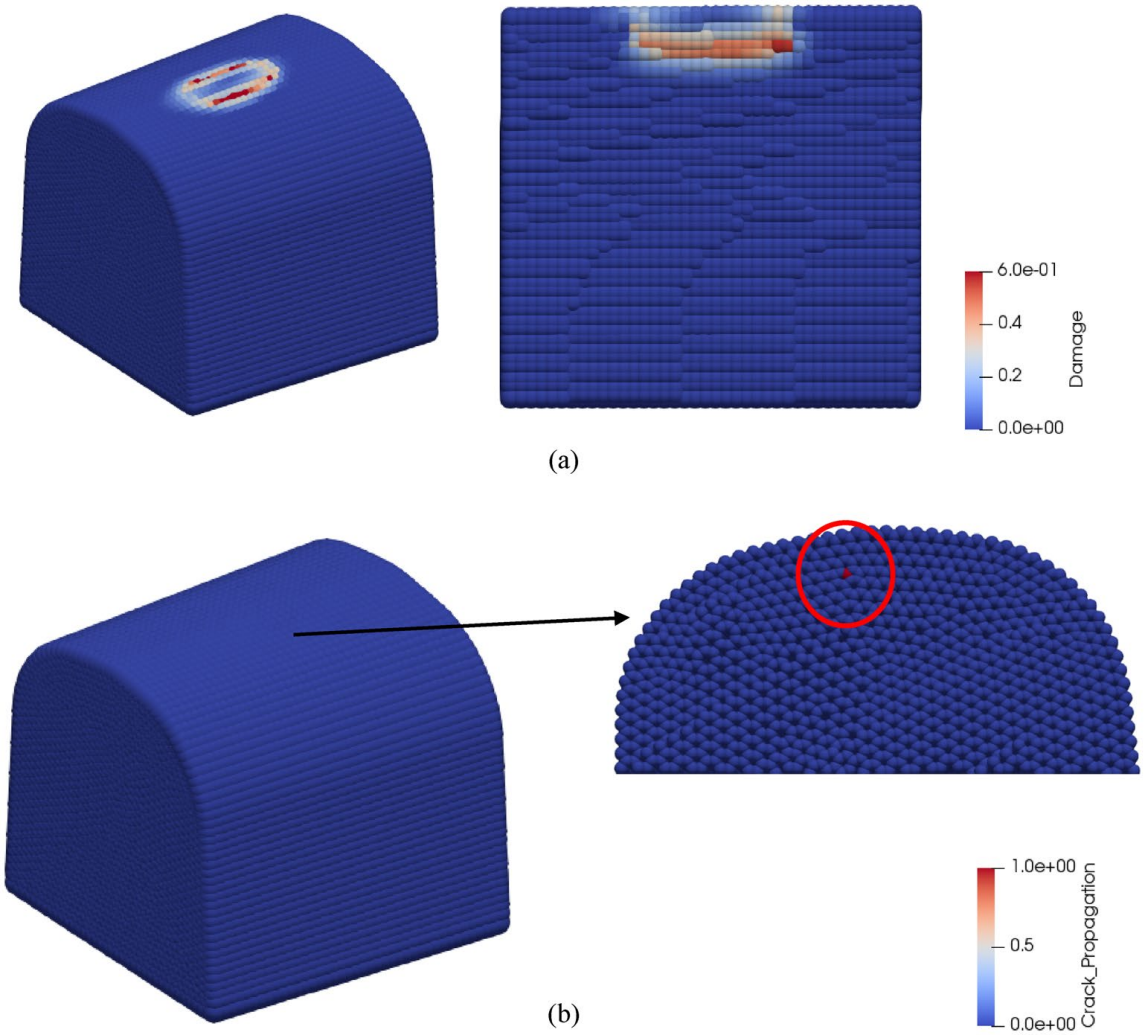
(**a**) Damage values over contact patch when a crack develops. (**b**) A cross-section of crossing nose that shows crack initiation.

In Fig. 15a, the damage values are illustrated for the cross-section 3 referring to the crossing nose sections in Fig. 2a, when a crack is initiated according to the crack-on-midplane method. Figure 15a also describes that the damage is concentrated beneath the contact surface and on the longitudinal boundaries of the contact surface. Despite high damage values, a crack is just initiated under the contact patch instead of longitudinal boundaries of the contact surface, as shown in Fig. 15b. It should be emphasized that the contact patch consists of multilayers since the force is distributed within the horizon. Furthermore, it is assumed that the bonds between the points within the contact area are not damaged in order to avoid instability. Hence, the contact volume acts like a flexible solid with no damage. That is also because the damage values for the points at the centre of the contact area are lower than other points within the contact area.

**Figure 16:**
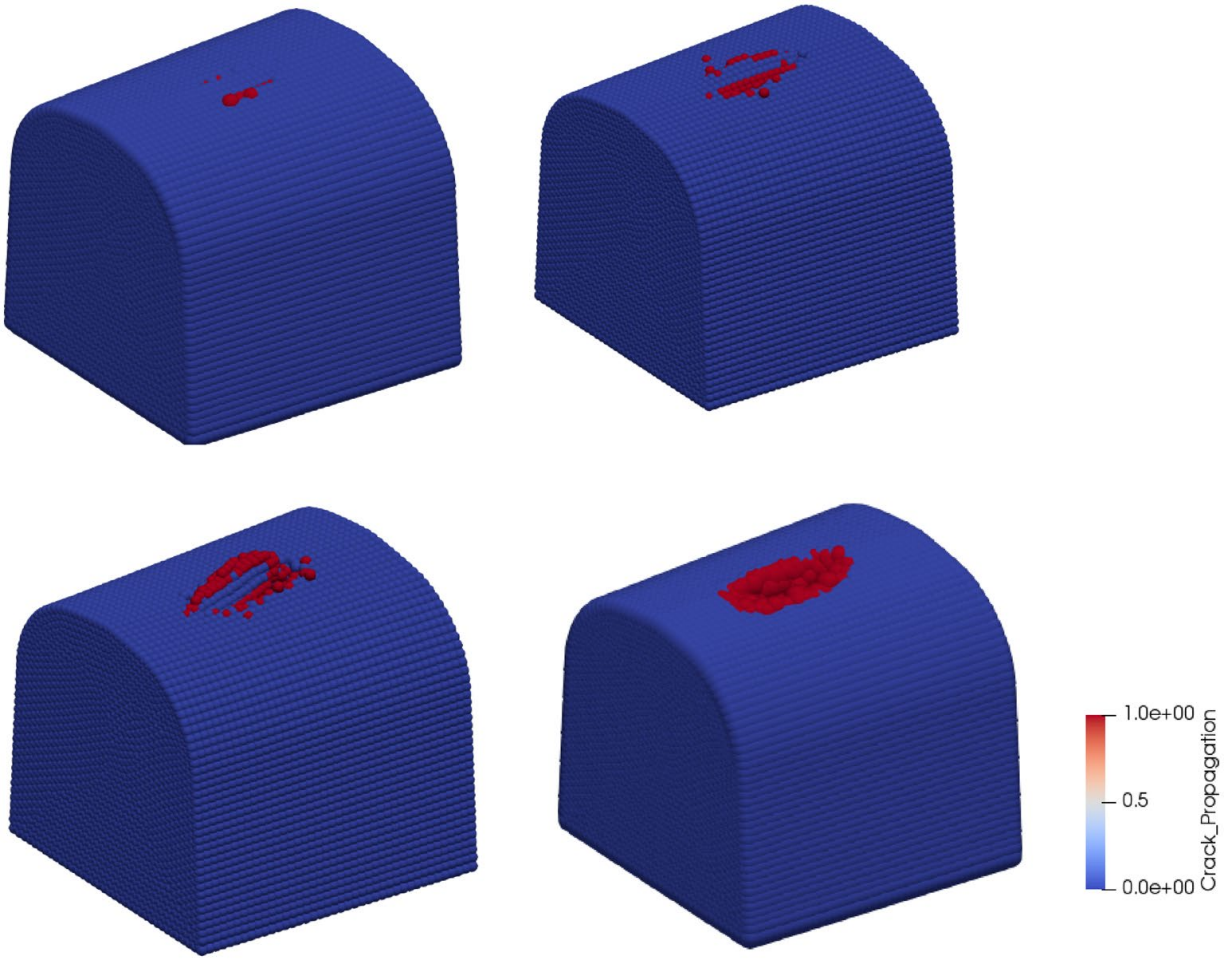
Development of a crack due to contact forces.

The developments of the crack from several cycles after initiation to complete failure are depicted in Fig. 16. From the figure, it can be seen that crack propagated from the sub-contact area to the surface of the crossing nose at multiple points. Then, it propagated in the longitudinal direction and led to a separation of contact patch from the crossing nose surface. Here, it is worth mentioning that some points in the pictures are larger than the others. Those larger points occur as a result of characteristic properties of the visualization tool and represent free nodes. A free node is the node having no bonds with other nodes. Finally, similar characteristics are observed for the other sections in terms of crack development and therefore, only the cross-section 3 is presented here.

**Figure 17:**
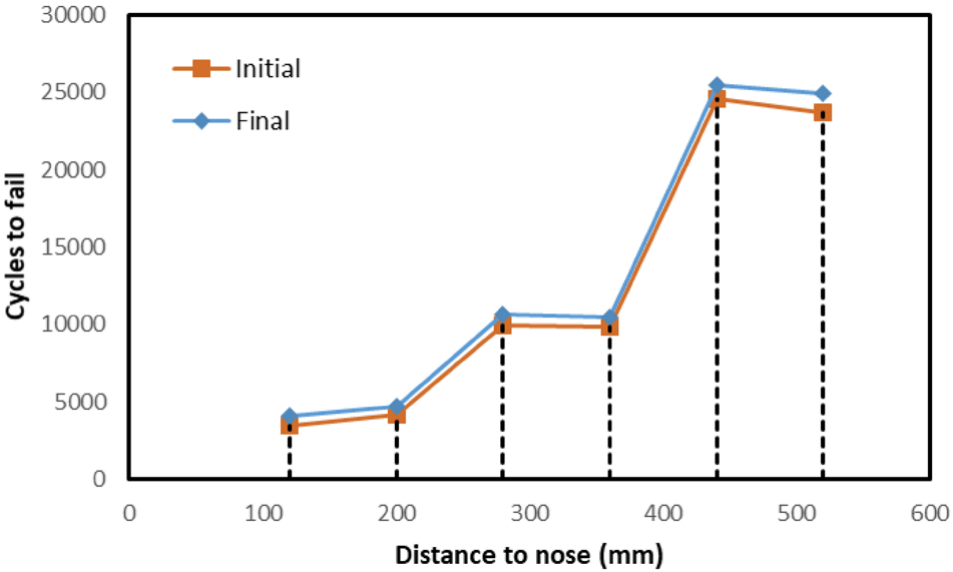
Fatigue lives of six different sections.

Figure 17 illustrates the life estimations from initiation to final failure stage for all sections. In comparison to reality, the accuracy of the estimations seems to be considerably low since the maximum cycle to failure is found to be 24.5 k cycles. The main reason behind such a low estimation will be explained in the discussion part. Nonetheless, Fig. 17 still presents significant outcomes. Firstly, the later the transition happens, the longer the crossing nose life is. The cross-sections 5-6 have 6 times higher life span in comparison to the cross-section 1. Another outcome presented in the figure is that the fatigue life of a crossing nose is significantly limited after crack initiation, which is consistent with the common knowledge^67^.

## Discussions

The fatigue model employed in this study has performed notably during validation simulations for crack initiation and propagation. Nonetheless, it has lost its effectiveness in the case of fatigue life estimation for the crossing nose sections. It has been also concluded that some challenges in PD theory must be firstly addressed in order to bring a solution to such above problems.

The first challenge would be related to the application of external forces. It has been observed in the fatigue model that an application of forces as a boundary condition on the surface of a crossing nose leads to a stability problem after some bonds are broken. In this study, it is called “Peridynamics Singularity”. Imagine two adjacent loading points close to crack surfaces. The closest one to crack surface loses more bonds when crack is developed. Since PD is associated with force equilibrium, the closest point will have more displacement and therefore, more strains. The higher strains will cause the collapse of all bonds of the closest one earlier and eventually, the closest one becomes a free node. Then, the second point will follow a similar process. Then one by one, all points will become free nodes. This process is a kind of erosion or explosion of the model. In the current study, to overcome that problem, the bonds within the contact area are disregarded in the damage calculation. Nevertheless, it has not eliminated the problem completely. This is perceptible in Fig. 15, where some free nodes can be seen.

It has been observed that the most important challenge is to exclude crack closing forces from the damage calculations since crack opening forces drive the crack propagation in most cases in reality. However, in the current simulation, crack closing forces negatively influence crack propagation and fatigue life. For instance, assume that there is a small crack that is aligned in parallel to the contact patch similar to the damaged area in Fig. 14a. In that case, vertical wheel loads would have a limited contribution in terms of crack opening as they try to close the crack. However, those forces support crack opening in the current simulation. In other words, if the direction of vertical contact force is reversed, then the same results will be obtained. To solve this challenge, many attempts have been done and all of them have failed. For instance, one of the attempts was to ignore compression forces during calculations. The attempt has failed due to non-locality that prevented the breaking of some bonds passing through crack planes as those bonds remained in compression. Another attempt was about considering only the certain directional components of the relative displacement vector. The attempt has failed as it is unlikely to decide the ratio of contribution in the case of bond rotation. Thereby, there is a need for a new fatigue model which can assess whether external forces are crack opening forces or not. Moreover, the contribution of compression forces into fatigue life must also be concerned. In conclusion, the current PD fatigue model seems to be suitable to simulate the problems where crack opening forces are dominant. PD model exhibited poor performance in calculation of total life of crossing nose of a railway turnout due to mixed loading conditions.

## Conclusions

In this paper, a novel investigation about fatigue damages over a crossing nose is presented. The investigation is conducted via the peridynamics fatigue damage model already available in the literature. The study also benefits from a novel damage assessment method proposed by the authors and additionally, introduces a new time-mapping method, which is a “must” in fatigue simulations due to long simulation times.

The fatigue model used in the current study is validated by FEM-PD comparison in terms of impact and quasi-static contact. It is found that PD theory slightly underestimates the displacements in comparison to FEM. The error is negligible in vertical loading whereas it is relatively higher in translational loading. The main error source is believed to be “surface effects”, which is prominent in PD. The mitigation method in Peridigm code is assumed to be sufficient for the scope of this study. Consequently, it is observed that PD theory yields a similar result with FEM. The proposed fatigue model, later, is validated by available experimental data in the literature. Both stages of the fatigue model are also considered.

To assess the fatigue damage over a turnout, several sections along a crossing nose are isolated and discretized. Then, contact patches are calculated explicitly with reference to Hertzian Contact Theory and contact loads are applied as a boundary condition in PD environment. In the current study, contact loads are distributed evenly over the contact patch and Coulomb’s friction is considered for lateral loads. The reason behind relatively simple definition of loads is that the available contact theories for PD applications are insufficient to consider the contact as a surface phenomenon. The magnitude of loads, the length of sections and material properties are specified with reference to the literature.

The outcomes of the study show that the fatigue model mimics the experiments in terms of crack propagation and fatigue life, accurately. Nevertheless, it shows poor performance while simulating the influence of contact forces on the fatigue life of a crossing nose of a railway turnout. Despite lower accuracy, simulations still provide some appreciable outcomes. The earlier transitions over a crossing nose decrease the total fatigue life, significantly. Furthermore, it has been found that the full failure occurs quickly following the crack initiation, which is also consistent with the findings in the related literature.

Finally and more importantly, the current study shows that PD is a promising tool to evaluate fatigue damage in structures, particularly large and dynamically forced structures such as crossing nose, although it suffers from several drawbacks such as high computational cost, limited inventory in terms of material, damage and contact models, and so on. Further advances are believed to facilitate the use of PD theory in terms of investigating long-term damages in railways.

## Data Availability

All data generated or analysed during this study are included in this published article.
